# SELENBP1 overexpression in the prefrontal cortex underlies negative symptoms of schizophrenia

**DOI:** 10.1073/pnas.2203711119

**Published:** 2022-12-13

**Authors:** Soojin Kim, Seong-Wook Kim, Mai Anh Thi Bui, Yeji Kim, Minsoo Kim, Jung-Cheol Park, Nam-Heon Kim, Gyeong Hee Pyeon, Yong Sang Jo, Jaewon Jang, Hae-Young Koh, Chae-Hong Jeong, Moonkyung Kang, Hyo Jung Kang, Yong-Woo Lee, Craig A. Stockmeier, Je Kyung Seong, Dong Ho Woo, Jung-Soo Han, Yeon-Soo Kim

**Affiliations:** ^a^Graduate School of New Drug Discovery and Development, Chungnam National University, Daejeon 34134, Republic of Korea; ^b^Research Center for Convergence Toxicology, Korea Institute of Toxicology, Daejeon 34114, Republic of Korea; ^c^Department of Biological Sciences, Konkuk University, Seoul 05029, Republic of Korea; ^d^School of Psychology, Korea University, Seoul 02841, Republic of Korea; ^e^Center for Neuroscience, Brain Science Institute, Korea Institute of Science and Technology, Seoul 02455, Republic of Korea; ^f^Department of Life Science, Chung-Ang University, Seoul 06911, Republic of Korea; ^g^Department of Biomedical Laboratory Science, Inje University, Gimhae 50834, Republic of Korea; ^h^Department of Psychiatry and Human Behavior, University of Mississippi Medical Center, Jackson, MS 39216; ^i^Korea Mouse Phenotyping Center, Seoul National University, Seoul 08826, Republic of Korea

**Keywords:** SELENBP1, schizophrenia, social behavior, frontal cortex, Brodmann area 9

## Abstract

The selenium-binding protein 1 (SELENBP1) has been reported to be up-regulated in the prefrontal cortex (PFC) of schizophrenia patients in postmortem reports. However, no causative link between SELENBP1 and schizophrenia has yet been established. Here, we provide evidence linking the upregulation of SELENBP1 in the PFC of mice with the negative symptoms of schizophrenia. We verified the levels of *SELENBP1* transcripts in postmortem PFC brain tissues from patients with schizophrenia and matched healthy controls. We also generated transgenic mice expressing human SELENBP1 (hSELENBP1 Tg) and examined their neuropathological features, intrinsic firing properties of PFC 2/3-layer pyramidal neurons, and frontal cortex (FC) electroencephalographic (EEG) responses to auditory stimuli. Schizophrenia-like behaviors in hSELENBP1 Tg mice and mice expressing *Selenbp1* in the FC were assessed. *SELENBP1* transcript levels were higher in the brains of patients with schizophrenia than in those of matched healthy controls. The hSELENBP1 Tg mice displayed negative endophenotype behaviors, including heterotopias- and ectopias-like anatomical deformities in upper-layer cortical neurons and social withdrawal, deficits in nesting, and anhedonia-like behavior. Additionally, hSELENBP1 Tg mice exhibited reduced excitabilities of PFC 2/3-layer pyramidal neurons and abnormalities in EEG biomarkers observed in schizophrenia. Furthermore, mice overexpressing *Selenbp1* in FC showed deficits in sociability. These results suggest that upregulation of SELENBP1 in the PFC causes asociality, a negative symptom of schizophrenia.

## Significance

Selenium-binding protein 1 (SELENBP1) is up-regulated in the prefrontal cortex of patients with schizophrenia as per postmortem reports, including the present study. However, no causative link between SELENBP1 and schizophrenia has yet been established. Here, we examined the anatomical deformities, physiological properties, electroencephalographic characteristics of the frontal cortex, and behaviors of animal models overexpressing human SELENBP1 to prove the role of SELENBP1 in schizophrenia pathogenesis. The animals exhibited several anatomical and electroencephalographic features of schizophrenia in the frontal cortex. Importantly, they showed behavioral endophenotypes related to the negative symptoms of schizophrenia as well as reduced sociability. These findings provide a causative link between PFC SELENBP1 upregulation and negative symptoms of schizophrenia.

Identifying the molecular changes causally involved in the pathogenesis of psychiatric diseases is a major challenge in neurobiological studies of mental disorders. Several studies probing genetic risk factors associated with schizophrenia have recently made advances along these lines, showing that the expression levels of the selenium-binding protein SELENBP1 (1) are altered in the brain and blood of patients with schizophrenia (2–6). A subsequent study analyzing gene expression in specific brain regions of patients with schizophrenia reported *SELENBP1* upregulation in the prefrontal cortex (PFC) (7).

Diagnostic symptoms of schizophrenia include negative symptoms characterized by social withdrawal, apathy, and emotional blunting (8, 9). Patients with schizophrenia with impaired social cognition show reduced volume of the right prefrontal white matter (10–14). The social impairments observed in patients with schizophrenia are similar to those in individuals with PFC damage (15). Consistent with this, similar deficient social behaviors have been observed in animals with PFC lesions (16) and in animals with a PFC knockdown of phospholipase C-β1 (PLC-β1), which is associated with the pathogenesis of schizophrenia (17). Therefore, the PFC may be a nexus for the negative symptoms of schizophrenia (18).

The etiology of schizophrenia has a significant genetic component (19–21). Genetic studies have investigated the potential causal role of susceptibility-related genes in the brain tissues of patients with schizophrenia by examining schizophrenia-like behaviors and neuropathological features of genetically modified animals (22). Neuregulin 1 (*Nrg1*)-deficient mice and transgenic (Tg) mice expressing dominant-negative Disc1 (disrupted in schizophrenia-1) exhibit pathological features similar to those found in the brains of patients with schizophrenia and behavioral phenotypes similar to those observed in animal models of schizophrenia (21, 23). However, there has been no causal link between SELENBP1 upregulation and the manifestation of diverse schizophrenia symptoms.

Here, we sought to address this issue. To replicate earlier finding on the SELENBP1 upregulation in the PFC region of patients with schizophrenia, we measured expression levels of *SELENBP1* transcripts in postmortem Brodmann area 9 (BA9) of patients with schizophrenia. We also generated and characterized Tg mice expressing human SELENBP1 (hSELENBP1), showing that these mice displayed brain correlates of schizophrenia and behavioral phenotypes characteristic of the negative symptoms of schizophrenia. To prove a causative link between overexpression of SELENBP1 in the PFC and social deficits, we generated and characterized a mouse model in which *Selenbp1* was transduced into the neonatal frontal cortex (FC).

## Results

### *SELENBP1* Upregulation in the BA9 Region of Patients with Schizophrenia.

**Fig. 1.**
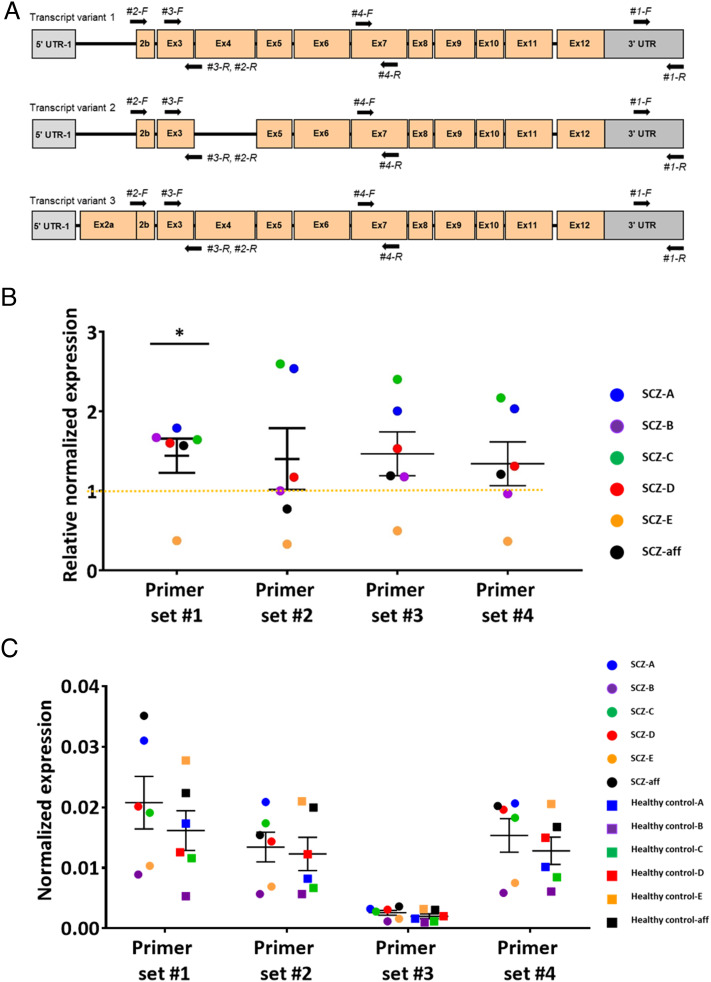
*SELENBP1* transcripts in the PFC of patients with schizophrenia. (*A*) Four *SELENBP1*-specific primer sets (*SI Appendix*, Table S1) targeting the 3′ UTR (set #1), exon 2 to 4 (set #2), exon 3 to 4 (set #3), and exon 7 (set #4) of human *SELENBP1* transcript variants. Ex, Exon; UTR, untranslated region. (*B*) Differences in *SELENBP1* transcripts levels in the PFC of five patients with schizophrenia (SCZ A–E; *SI Appendix*, Table S2) and one with schizoaffective disorder (SCZ-aff; *SI Appendix*, Table S2) relative to that in six matched healthy controls (Healthy A–F; *SI Appendix*, Table S2), measured using primer set #1 (*P* < 0.05), set #2 (*P* = 0.17), set #3 (*P* = 0.08), and set #4 (*P* = 0.13). *SELENBP1* expression in matched healthy controls (Healthy A–F, *SI Appendix*, Table S2) was given a fold-change value of one, indicated by the horizontal dotted yellow line (*P < 0.05). (*C*) Normalized expression values in patients with schizophrenia and matched healthy controls. The normalized expression values were subjected to the 2^ΔΔCt^ method to obtain the relative normalized expression value in Fig. 1*B*. Data are presented as means ± SEM.

Previous postmortem brain studies have revealed the upregulation of *SELENBP1* in the BA9 region of patients with schizophrenia (4–7, 24). To confirm these findings, we collected postmortem BA9 samples from six patients with schizophrenia (SCZ A–E), one patient with schizoaffective disorder (SCZ-aff) and five healthy controls (Healthy A–F), matched by sex, race, age, postmortem interval (PMI), and tissue pH (Table 1). The *SELENBP1* transcript levels in these six matched pairs of human brain samples were measured by quantitative reverse transcription-PCR using four sets of *SELENBP-*specific primers targeting the 3′ untranslated region (set #1), exon 2 to 4 (set #2), exon 3 to 4 (set #3), and exon 7 (set #4) (Fig. 1*A* and *SI Appendix,* Table S1). These analyses revealed alterations, mostly upregulation, in *SELENBP1* expression in BA9 tissues from SCZ A–E and patients with SCZ-aff compared to those from healthy A–F individuals (Fig. 1 *B* and *C* and *SI Appendix,* Table S2). The relative expression levels of *SELENBP1* transcripts in patients with schizophrenia were higher than those in controls (primer set #1, t t_5_ = 2.05, *P* < 0.05, one-sample *t* test, one-tailed, Fig. 1*B*), consistent with previous findings (4, 7). These results confirm that *SELENBP*1 levels are up-regulated in the BA9 region of patients with schizophrenia. We also analyzed copy number variations (CNVs) in two *SELENBP1*-overexpressing PFC samples and found a partial overlap of these CNVs with the CNVs of long non-coding RNAs (lncRNAs) of autism spectrum disorder (ASD)-related genes (*SI Appendix,* Table S3).

**Table 1.** Postmortem human PFC samples from subjects with psychosis and non-psychiatric controls

Pair	Subject	Age (y)	Sex	Race	PMI	Tissue pH	Diagnosis
A	SCZ-A	40	M	White	12	6.47	Schizophrenia paranoid type
	Healthy-A	37	M	White	17	6.47	No diagnosis
B	SCZ-B	43	M	White	16	6.77	Schizophrenia
	Healthy-B	44	M	White	24	6.65	No diagnosis
C	SCZ-C	44	M	Black	21.2	6.56	Schizophrenia paranoid type
	Healthy-C	46	M	Black	11	6.95	No diagnosis
D	SCZ-D	47	M	Black	16	6.62	Schizophrenia chronic disorganized
	Healthy-D	46	M	Black	19	6.95	No diagnosis
E	SCZ-E	41	M	Black	16.5	6.02	Schizophrenia paranoid type
	Healthy-E	35	M	Black	21	6.26	No diagnosis
F	SCZ-aff	42	M	White	24	7.14	Schizoaffective disorder
	Healthy-F	43	M	White	23	6.49	No diagnosis

SCZ, subjects with schizophrenia; SCZ-aff, subjects with schizoaffective disorder; healthy, non-psychiatric control subjects; M, male; PMI, postmortem interval (h).

### Higher Levels of SELENBP1 and Neuroanatomical Defects in the Brain Regions of hSELENBP1 Tg Mice.

We generated Tg mice carrying a human SELENBP1 gene regulated by the chicken β-actin promoter and genotyped Tg founders and their progenies by PCR using a specific set of primers that detected transgene integration (*SI Appendix,* Fig. S1*A*). A 733-bp PCR product was detected in genomic DNA samples extracted from the tails of Tg mice but not in those from non-Tg mice (*SI Appendix,* Fig. S1*B*). In addition to increased levels of *SELENBP1* transcripts, we found higher levels of SELENBP1 protein in primary tissues and PFC of Tg mice than in non-Tg mice (PFC, t_7_ = −36.89, *P* < 0.001; muscle, t_7_ = −3.17, *P* < 0.05; pancreas, t_7_ = −8.25, *P* < 0.001; skin, t_7_ = −5.25, *P* < 0.01; unpaired *t* test; *SI Appendix,* Fig. S1*C*). Immunohistochemical staining of the mouse brain showed elevated levels of SELENBP1 in multiple brain regions, including the PFC and hippocampus, in Tg mice than in non-Tg littermates (*SI Appendix,* Fig. S1*D*). Interestingly, the cortices were malformed in hSELENBP1 Tg mice. Specifically, cortical thickness was significantly reduced in hSELENBP1 Tg mice than non-Tg mice (t_16_ = 2.33, *P* < 0.05; unpaired *t* test; *SI Appendix,* Fig. S2 *A* and *B*). Furthermore, some SELENBP1 Tg mice (#439 and #473) exhibited heterotopias or ectopias (*SI Appendix,* Fig. S2*C*) caused by inappropriate migration through the marginal zone, resulting in a lack of layer I (*SI Appendix,* Fig. S2*C*) without exhibiting any other severe brain developmental abnormalities (*SI Appendix,* Fig. S2*D*).

### Reduced Excitability of Layer 2/3 Pyramidal Neurons in the PFC of hSELENBP1 Tg Mice.

**Fig. 2.**
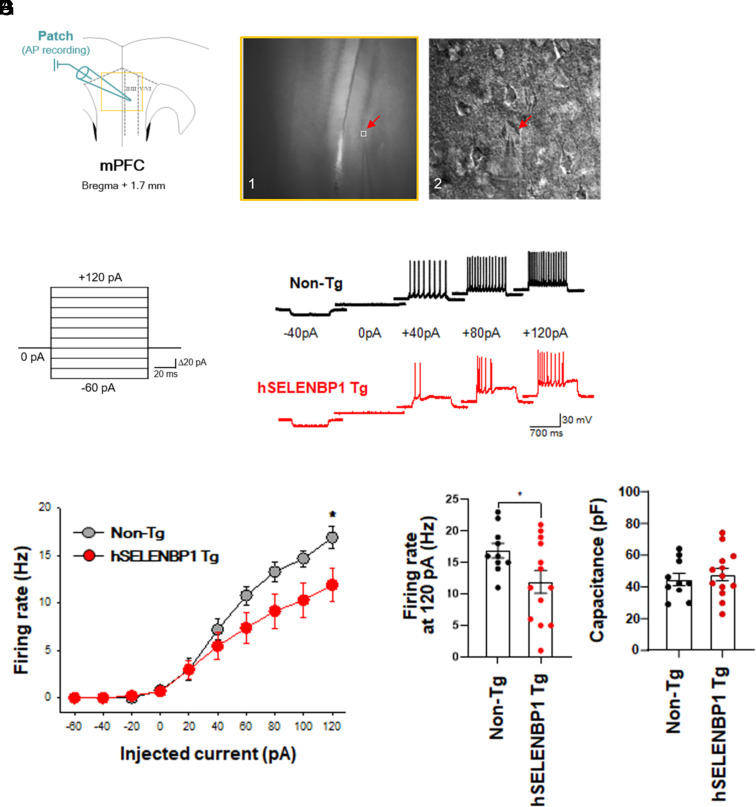
Decreased excitability of 2/3-layer pyramidal neurons in the PFC of Tg mice expressing human SELENBP1 (hSELENBP1). (*A*) Patch-clamp recording scheme for measuring intrinsic firing properties of neurons in coronal sections. (*B*) The captured image of patch-clamp recording of pyramidal neurons. The *Left* panel is a lower magnification image of the yellow quadrangle in (*A*), and the *Right* panel is a higher magnification image of the white quadrangle in the *Left* panel. (*C*) Current-clamp protocol. (*D*) Representative firing traces of pyramidal neurons from non-Tg (*Upper*) and hSELENBP1 Tg (*Lower*) mice at different current pulses. (*E*) Averaged number of action potential of pyramidal neurons from non-Tg (n = 10) and hSELENBP1 Tg (n = 13) mice (*P < 0.05). (*F*) Diminished firing rates of pyramidal neurons from hSELENBP1 Tg mice compared with those of non-Tg mice (*P < 0.05). (*G*) No difference in membrane capacitance between the two genotypes. Data are presented as means ± SEM.

To evaluate the functional integrity in the PFC of hSELENBP1 Tg mice, we measured the intrinsic firing properties of layer 2/3 pyramidal neurons in PFC brain slices from non-Tg and hSELENBP1 Tg mice (Fig. 2 *A* and *B*). To this end, we injected depolarizing currents (20-pA increments, 10 steps, 1-s duration) into layer 2/3 pyramidal neurons in the PFC from a resting membrane potential of –60 mV (Fig. 2*C*) and compared the activity between the two groups. A two-way repeated-measures (RM) ANOVA of the frequency of action potentials (group, non-Tg vs. SELENBP1 Tg; repeated variable, injected current) revealed no effect of group (F_1,21_ = 2.27, *P* = 0.15) but showed a significant effect of current (F_1,21_ = 135.65, *P* < 0.001); it also showed a significant interaction between effects of group and current (F_9,189_ = 3.79, *P* < 0.001; Fig. 2 *D* and *E*). Post hoc analyses showed that neuronal excitability was reduced at +120 pA (Fig. 2*F*). No difference in the membrane capacitance of the recorded neurons was detected between the two groups (*t*_21_ = 0.54, *P* = 0.60; Fig. 2*G*). Thus, these results indicate that SELENBP1 upregulation in the FC during schizophrenia pathogenesis leads to a reduction in neuronal excitability.

### Abnormal FC Electroencephalographic (EEG) Responses to Paired Auditory Stimuli in the hSELENBP1 Tg Mice.

**Fig. 3.**
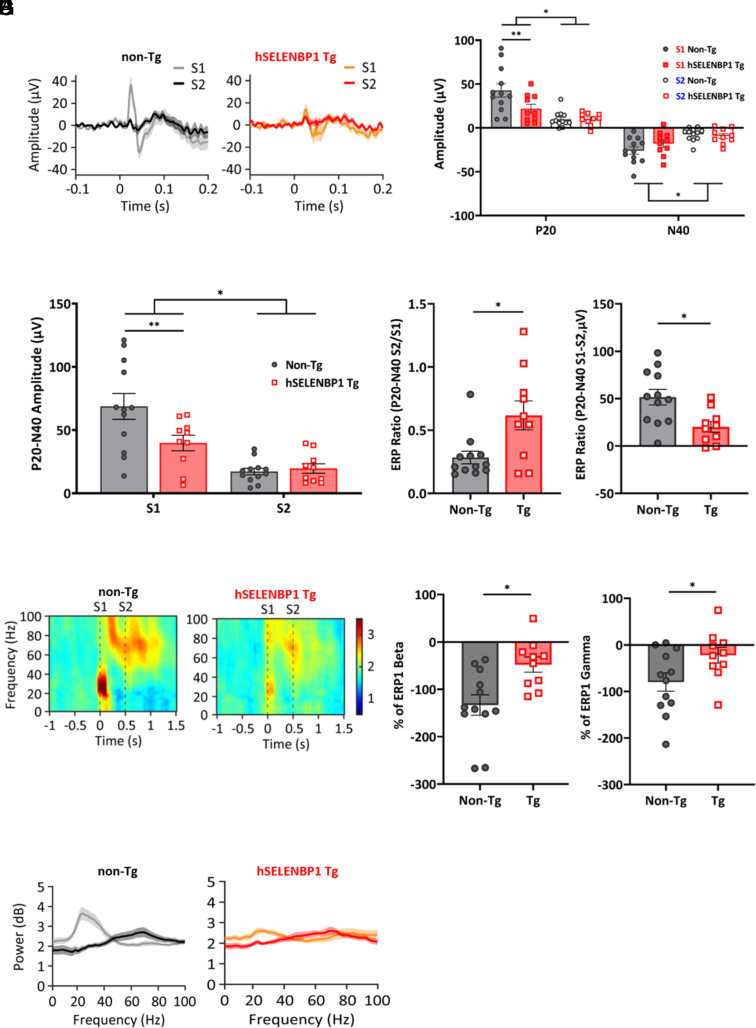
FC ERP in the non-Tg and hSELENBP1 mice. (*A*) The FC EEGs of the non-Tg (n = 12) and hSELENBP1 (n = 10) mice were measured by averaging the ERPs of 100 repetitions of two identical 5 kHz 50 ms tones (S1 and S2) with 50 ms interstimulus interval. (*B* and *C*) Animals in two groups exhibited significantly larger P20, N40, and P20—N40 in S1 than in S2 (*). hSELENBP1 Tg mice had smaller P20 and P20—N40 amplitudes in S1 than non-Tg mice (**). (*D* and *E*) Significant between-group differences in normalized ratios S2/S1 and S1-S2 (*). (*F*) FC grand average spectrograms. (*G* and *H*) Less reduction in beta and gamma power of the hSELENBP1 Tg mice from S1 to S2 compared to non-Tg mice (*). (*I*) No between-group differences in S1 (ERP1) and S2 (ERP2) across the entire frequency range. Data are presented as means ± SEM.

We also measured the FC grand average event-related potentials (ERPs) generated by paired tones (S1 and S2) to examine the functional integrity of hSELENBP1 Tg mice (Fig. 3*A*). A two-way RM ANOVA of the P20 (group: F_1,20_ = 3.19, *P* = 0.09; P20, F_1,20_ = 26.57, *P* < 0.001; interaction, F_1,20_ = 6.09, *P* < 0.05) and P20–N40 amplitudes (group, F_1,20_ = 2.88, *P* = 0.11; P20-N40, F_1,20_ = 47.82, *P* < 0.001; interaction, F_1,20_ = 9.11, *P* < 0.01) revealed that both non-Tg mice and hSELENBP1 Tg mice had significantly larger P20 and P20–N40 amplitudes in S1 than in S2 (Fig. 3 *B* and *C*). Subsequent post hoc analyses showed that hSELENBP1 Tg mice had smaller P20 and P20–N40 amplitudes in S1 than non-Tg mice (Fig. 3 *B* and *C*). A two-way RM ANOVA of the N40 (group, F_1,20_ = 0.55, *P* = 0.47; N40, F_1,20_ = 29.23, *P* < 0.001; interaction, F_1,20_ = 3.11, *P* = 0.06) revealed that both non-Tg mice and hSELENBP1 Tg mice had significantly larger N40 in S1 than in S2 (Fig. 3*B*). Unpaired *t* tests of normalized ratios S2/S1 (*t*_20_ = −2.82, *P* < 0.01) and S1−S2 (*t*_20_ = 3.02, *P* < 0.01) showed significant between-group differences (Fig. 3 *D* and *E*).

Next, we examined whether there were between-group differences in evoked beta (15 to 25 Hz) and gamma power (26 to 50 Hz) during S1 (0 to 0.05 s) and S2 (0.5 to 0.55 s) (Fig. 3*F*). The beta (*t*_20_ = 3.07, *P* < 0.01) and gamma (*t*_20_ = 2.17, *P* < 0.05) powers of the hSELENBP1 Tg mice were less significantly reduced from S1 to S2 compared to those of non-Tg mice (Fig. 3 *G* and *H*). No between-group differences in S1 (ERP1) and S2 (ERP2) were found across the entire frequency range (2 to 100 Hz) (*t*_20_ < 0.67, *P* > 0.51, Fig. 3*I*). Most EEG responses of the parietal cortex exhibited no between-group differences and different characteristics from those of the FC (*SI Appendix,* Fig. S3).

### hSELENBP1 Tg Mice Exhibit Asociality, Nesting Behavior Deficits, and Reduced Sucrose Consumption.

**Fig. 4.**
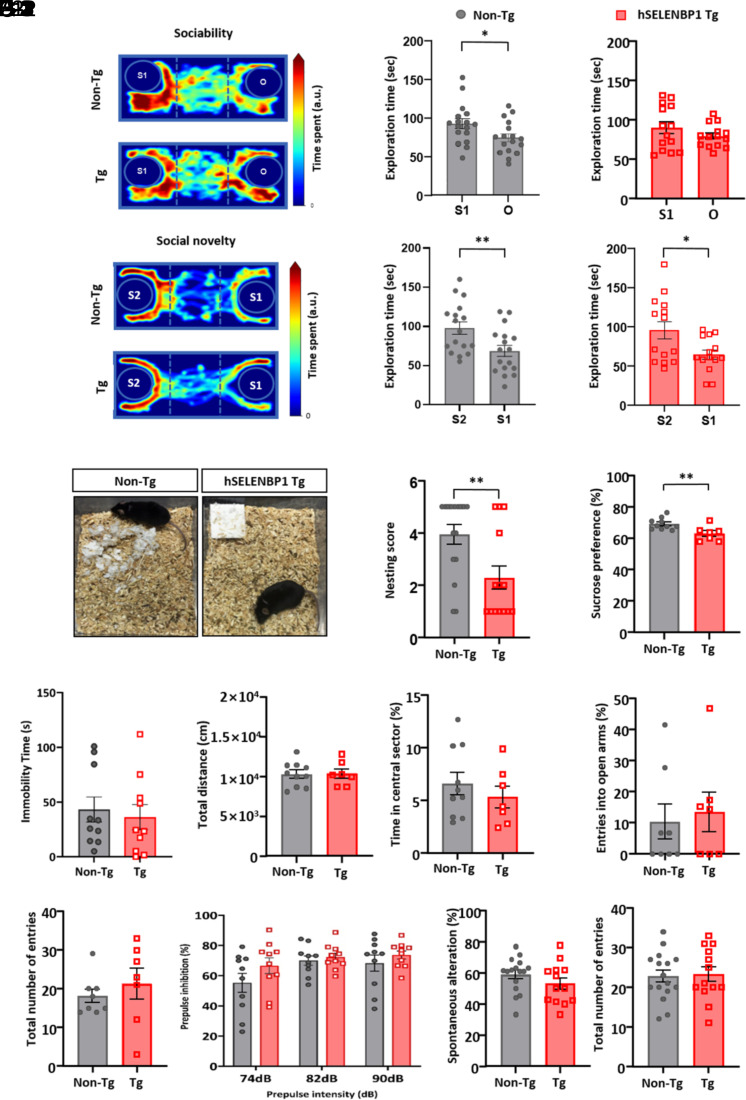
Asociality and anhedonia of hSELENBP1 Tg mice. (*A*) Time spent in the three chambers of the social approach task, depicted as a pseudo-colored heat map (*A*-1). Non-Tg mice explored stranger 1 (S1) more than an object (O) (*A-*2), but the hSELENBP1 Tg mice did not (*A-*3). (*B*) Time spent in the three chambers of the social novelty task, represented as a pseudo-colored heat map (*B*-1). Both non-Tg (*B-2*) and hSELENBP1 Tg (*B*-3) mice explored S2 more than S1. (*C*) Representative pictures of cages of non-Tg (*C*-1) and hSELENBP1 Tg (*C*-2) mice, showing impaired nesting behaviors of hSELENBP1 Tg mice. (*D*) Absence of sucrose preference in hSELENBP1 Tg mice. (*E*) Immobility times in the FST showed no between-group differences. (*F*) No between-group differences in the total distance of spontaneous locomotion (*F*-1) or percentage of time in the central sector (*F*-2) in the open-field task. (*G*) No between-group differences in the percentage of entries into open arms (*G*-1) or the total number of entries (*G*-2) in the elevated plus-maze test. (*H*) No between-group differences in the percentage of prepulse inhibition. (*I*) No between-group differences in the percentage of spontaneous alternation (*I*-1) or the total number of arm entries (*I*-2). *P < 0.05, **P < 0.01. a.u., arbitrary units.

We next investigated the schizophrenia-like endophenotypes of hSELENBP1 Tg mice, first evaluating the social behaviors of mice using a three-chamber social approach and novelty task. In the social approach task (Fig. 4*A*-1), as measured by exploration time, non-Tg mice preferred to explore the novel mouse (stranger 1) over an inanimate object (*t*_16_ = 2.44, *P* < 0.05; paired *t* test; Fig. 4*A*-2). In contrast, hSELENBP1 Tg mice did not exhibit this behavior (*t*_13_ = 1.25, *P* = 0.23; paired *t* test; Fig. 4*A*-3), indicating sociability deficits. In the social novelty task (Fig. 4*B*-1), when the inanimate object was replaced with another novel mouse (stranger 2), both non-Tg (Fig. 4*B*-2) and hSELENBP1 Tg (Fig. 4*B*-3) mice preferred stranger 2 to stranger 1 (non-Tg: *t*_16_ = 4.65, *P* < 0.001; Tg: *t*_13_ = 3.50, *P* < 0.01; paired *t* test), indicating that social novelty recognition was intact in Tg mice. We further found that hSELENBP1 Tg mice exhibited impairment in nesting behavior (Fig. 4*C*-1), a behavioral measure of schizophrenia-like social withdrawal (25, 26) (*t*_28_ = 2.86, *P* < 0.01; independent *t* test; Fig. 4*C*-2). Specifically, whereas non-Tg mice built an identifiable nest at a distinct location in the cage, hSELENBP1 Tg mice did not form nests and tended to scatter pieces of nesting material over the cage floor (Fig. 4*C*-2). Tests of sucrose preference showed that hSELENBP1 Tg mice consumed less sucrose than non-Tg mice (independent *t* test*, t*_15_ = 2.98, *P* < 0.01; Fig. 4*D*), indicating anhedonia in Tg mice. In the forced swim test (FST), adapted mostly to assess the depressive-like behavior, no between-group difference was found (independent *t* test*, t*_18_ = −0.43, *P* = 0.67; Fig. 4*E*)

Hyperlocomotion is a positive symptom of schizophrenia in humans and rodents (8, 9). Since the deficits observed in the social approach tasks might be caused or influenced by anxiety, we measured anxiety-like behaviors in hSELENBP1 Tg mice using open-field and elevated plus-maze tasks. In the open-field task, hSELENBP1 Tg and non-Tg mice showed no significant difference in locomotor activity (*t*_15_ = −0.10, *P* = 0.92; independent *t* test; Fig. 4*F*-1) and did not differ in the percentage of time spent in the central area of the open-field arena (*t*_15_ = 0.33, *P* = 0.75; independent *t* test; Fig. 4*F*-2). Similarly, in the elevated plus-maze task, we found no significant between-group differences in the percentage of entries into the open arms (*t*_13_ = −0.37, *P* = 0.72; independent *t* test; Fig. 4*G*-1) or the total number of arm entries (*t*_13_ = −1.02, *P* = 0.33; independent *t* test; Fig. 4*G*-2). Additionally, we examined sensorimotor gating status of hSELENBP1 Tg mice using the prepulse inhibition task and found no between-group differences (group, F_1,18_ = 1.63, *P* = 0.22; intensity, F_2,36_ = 6.18, *P* < 0.01; interaction, F_2,26_ = 0.94, *P* = 0.40; Fig. 4*H*). An assessment of working memory using the Y-maze task showed no differences in the percentage of spontaneous alternations between hSELENBP1 Tg mice and non-Tg mice (*t*_29_ = 1.30, *P* = 0.20; independent *t* test; Fig. 4*I*-1) or the total number of arm entries (*t*_29_ = 0.10, *P* = 0.93; independent *t* test; Fig. 4*I*-2). These results demonstrate no effects of h*SELENBP1* on locomotion, working memory, or anxiety-like behavior.

### Overexpression of *Selenbp1* in the FC Causes Sociability Deficits in Mice.

Before examining the behavioral effects of modulating *Selenbp1* expression in the FC, we measured the expression levels of endogenous *Selenbp1* in the cortex region of wild-type C57B/6 mice. These experiments showed that *Selenbp1* expression in the mouse brain was high in neonates (2-d-old mice) and low thereafter (*SI Appendix,* Fig. S4*A*). To examine whether the behavioral phenotypes of negative symptoms observed in hSELENBP1 Tg mice depend on the FC, we injected lentiviral vectors expressing recombinant mouse *Selenbp1* or *DsRed2* into the FC of 2-d-old wild-type mice (*SI Appendix,* Fig. S4 *B* and *C*). We confirmed the FC expression levels of transduced and endogenous *Selenbp1* transcripts in mice injected into the FC with the *Selenbp1*-encoding lentiviral vector (*SI Appendix,* Fig. S4*D*).

**Fig. 5.**
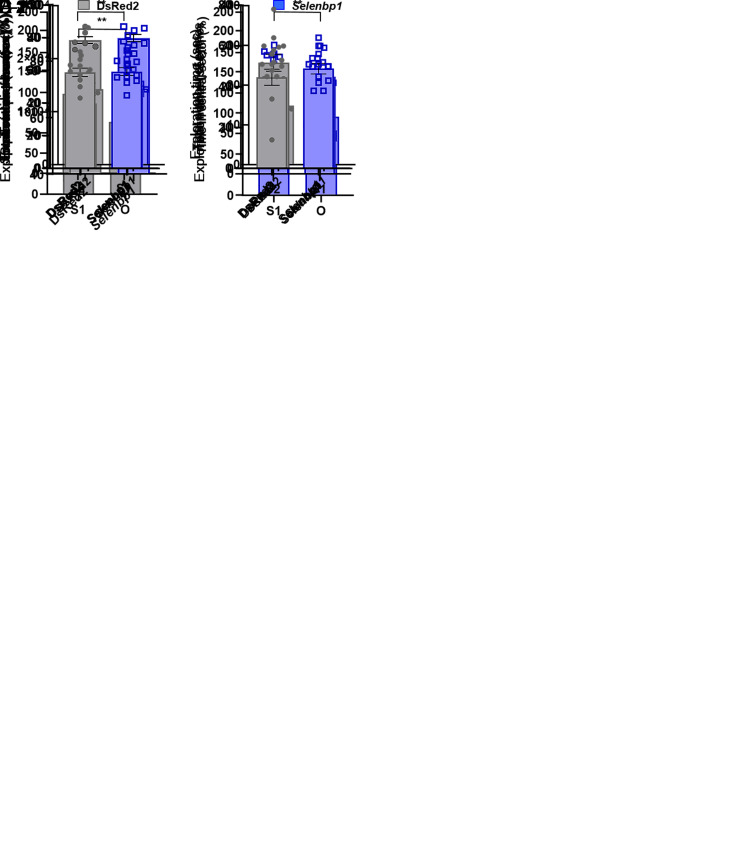
Deficits in the sociability of mice injected into the FC with a lentiviral vector encoding *Selenbp1*. (*A*) Normal social approach behaviors in control DsRed2 mice (*A*-1), but not Selenbp1 mice (*A*-2) in the three-chamber social approach task. (*B*) Preference for S1 over S2 by both DsRed2 (*B*-1) and *Selenbp*1 (*B*-2) in the three-chamber social novelty task. (*C*) No between-group differences in sucrose preference. (*D*) No between-group differences in the total distance of spontaneous locomotion (*D*-1) or percentage of time in the central sector of the open-field task (*D*-2). (*E*) No between-group differences in the percentage of entries into open arms (*E*-1) or the total number of entries into each arm (*E*-2) in the elevated plus-maze test. (*F*) No between-group differences in the percentage of correct spontaneous alternations (*F*-1) or the total number of entries into each arm (*F*-2) in the Y-maze task. Data are expressed as means ± SEM.

The social approach behavior of mice with FC-transduced *Selenbp1* was examined using the three-chamber task and compared to control *DsRed2* mice. In the social approach session, *DsRed2* control mice showed a preference (exploration time) for stranger 1 compared to the object (*t*_12_ = 2.76, *P* < 0.05; paired *t* test; Fig. 5*A*-1). However, no preference for stranger 1 was observed in mice with FC-transduced *Selenbp1* (*t*_14_ = 1.596, *P* = 0.14; paired *t* test; Fig. 5*A*-2), indicating that mice with FC-transduced *Selenbp1* were asocial. In the social novelty session, when the object was replaced with stranger 2, both *DsRed2*-injected (*t*_12_ = −3.53, *P* < 0.01; paired *t* test; Fig. 5*B*-1) and *Selenbp1*- (*t*_14_ = −4.41, *P* < 0.01; paired *t* test; Fig. 4*B*-2) mice preferred stranger 2 to stranger 1, indicating that social novelty recognition remained intact in mice with FC-transduced *Selenbp1*.

In contrast to the diminished preference for sucrose observed in hSELENBP1 Tg mice than in non-Tg mice, mice with FC-transduced *Selenbp1* showed no differences in sucrose preference compared to control *DsRed2* mice (*t*_11_ = −0.35, *P* = 0.74; independent *t* test*;* Fig. 5*C*). There were no between-group differences in the total distance of spontaneous locomotion (independent t test*, t*_27_ = −0.13, *P* = 0.90; Fig. 5*D*-1) or the percentage of time spent in the central sector of the open-field task (*t*_27_ = −1.13, *P* = 0.27; independent *t* test; Fig. 5*D*-2). Moreover, the number of entries into each arm (*t*_24_ = 1.51, *P* = 0.15; independent *t* test; Fig. 5*E*-1) and percentage of entries into the open arms in the elevated plus-maze test (*t*_24_ = −1.21, *P* = 0.24; independent *t* test; Fig. 5*E*-2) did not differ between the groups. Finally, there were no between-group differences in the percentage of spontaneous alternations (*t*_19_ = −0.15, *P* = 0.89; independent *t* test; Fig. 5*F*-1) or the total number of arm entries (*t*_19_ = −0.91, *P* = 0.37; independent *t* test; Fig. 5*F*-2) in the Y-maze spontaneous alternation task. Additionally, we examined the attentional status of *DsRed2* controls and mice with FC-transduced *Selenbp1* mice using the attentional set-shifting task (ASST), a behavioral task for measuring cognitive symptoms in schizophrenia animal models (27). All mice in both groups showed comparable performances in all discrimination sessions, except for one discrimination session (*SI Appendix*). Specifically, *DsRed2 controls* and mice with FC-transduced *Selenbp1* showed no significant differences in the number of trials to reach the criterion in simple discrimination (t_26_ = 0.09, *P* = 0.92), compound discrimination (CD; t_26_ = 0.31, *P* = 0.76), CD reversal (t_26_ = 0.47, *P* = 0.64), intra-dimensional shift 1 (IDS1) discrimination (t_26_ = 0.36, *P* = 0.72), IDS1 reversal (t_26_ = −0.77, *P* = 0.45), IDS2 reversal (t_26_ = −1.51, *P* = 0.14), and extradimensional shift discrimination (t_26_ = −0.75, *P* = 0.46). However, mice with FC-transduced *Selenbp1* required significantly more trials to reach the criterion in the IDS2 session than *DsRed2* controls (t_26_ = −2.29, *P* < 0.05). See *SI Appendix*, Fig. S5 for the details. Thus, mice with FC-transduced *Selenbp1* exhibited normal behaviors in the examined behavioral tasks, except for impairments in the social approach and the intra-dimensional shift session of the ASST.

## Discussion

Postmortem neurochemical investigations of patients with schizophrenia, including those reported in the present study, imply an association between increased PFC *SELENBP1* expression and schizophrenia development. However, no study has demonstrated a causal genetic link between increased PFC *SELENBP1* expression and schizophrenia. To provide experimental evidence for such a causal relationship, we generated Tg mice carrying h*SELENBP1* and transduced mouse *Selenbp1* into the FC of wild-type mice. hSELENBP1 Tg mice exhibited neuroanatomical correlates, a decreased excitability of putative glutamatergic neurons in the PFC, impaired sensory gating of the FC, and negative behavioral endophenotypes of schizophrenia. We also observed a deficit in the social approach task and IDS discrimination of ASST in mice with FC-transduced *Selenbp1*.

To determine the relationship between increased *SELENBP1* expression and schizophrenia-like endophenotypes, we generated Tg mice carrying human SELENBP1 and characterized them neuroanatomically, electroencephalographically, and behaviorally. hSELENBP1 Tg mice exhibited thinner cortices, heterotopias, and ectopias, all of which are observed in the brains of patients with schizophrenia (28, 29). We also measured the frequency of action potentials evoked by depolarizing currents in PFC 2/3-layer pyramidal neurons of hSELENBP1 Tg mice and found a decreased excitability of these neurons. Additionally, the FC ERPs to a paired tone presentation, a translational endophenotype for schizophrenia (30), were measured in the hSELENBP1 Tg mice. hSELENBP1 Tg mice had more minor ERP amplitudes and evoked beta and gamma power than non-Tg mice, indicating auditory sensory gating impairment. Finally, we extensively examined the behavioral endophenotypes of hSELENBP1 Tg mice using several behavioral tasks, showing that these mice exhibited schizophrenia-like negative phenotypes: less social preference in the three-chambered social approach, task deficits in nesting behavior, and the absence of sucrose preference. However, hSELENBP1 Tg mice exhibited no differences compared to non-Tg mice in other behaviors relevant to schizophrenia-like endophenotypes, including locomotion, social novelty, sensorimotor gating, and working memory. We also found no difference between hSELENBP1 and non-Tg mice in the FST. Interestingly, abnormalities in the FC ERPs were reported in serine racemase knockout (SRKO) mice, a genetic mouse model of N-methyl-D-aspartate (NMDA) receptor hypofunction (31). SRKO mice exhibit altered glutamatergic neurotransmission and a deficit in the social novelty task but no deficits in sensorimotor gating (31, 32). Multiple similarities between SRKO and hSELENBP1 Tg mice suggest reduced sociality that occurs with NMDA hypofunction in the PFC. However, further studies are required to examine the integrity of GABAergic neurons and molecular changes in the PFC of hSELENBP1 Tg mice.

Numerous studies have implicated PFC dysfunction or hypofunction in the negative symptoms of schizophrenia, including impaired social behavior (10–14, 33). Consistent with this, social behavior is impaired by PFC lesions in mice (16) and PFC dysfunction in mice and humans (34, 35). Notably, the social impairments of patients with schizophrenia are similar to patients with PFC damage (15). PLC-β1 is involved in postnatal-cortical development and neuronal plasticity (36), and mice lacking PLC-β1 exhibit schizophrenia endophenotypes (37). Our previous studies showed that the knockdown of PLC-β1 in the PFC of the mice disrupted social behavior (17, 38). Here, we sought to determine whether *Selenbp1* transduction in the FC impaired social behavior. First, to determine the optimal mouse age for injecting a lentiviral vector encoding *Selenbp1*, we assessed the age-dependent expression of endogenous SELENBP1 in the cortex of the mouse brain (*SI Appendix,* Fig. S4*A*). Endogenous SELENBP1 expression was high on postnatal days 2 and 7, with little expression after that. Based on these observations and previous reports that injection of nerve growth factor into the neonatal FC induces schizophrenia-like endophenotypes in adults (39), we injected LV-mCMV-*Selenbp1* into the FC of postnatal mice on day 2. This experimental intervention disrupted social and attentive behaviors; however, the other behaviors examined remained intact. Therefore, further studies are needed to elucidate the relationship between expression levels of SELENBP1 or SELENBP1-associated markers in the FC and the degree of social impairment. Notably, a neonatally treated or lesioned animal might be an optimal neurodevelopmental model of schizophrenia since schizophrenia is the end state of abnormal neurodevelopment-initiated years before the onset of brain disorders (40–42).

As exemplified here with hSELENBP1 Tg mice, this mouse model is a valuable tool for performing multiple-level analyses to link SELENBP1 overexpression in the brain to specific anatomical, electrophysiological, electroencephalographical, and behavioral outcomes. However, as described above, endogenous SELENBP1 expression was high during the postnatal period, with little expression after that. In addition, *SELENBP1* mRNA levels are increased in the PFC of patients with schizophrenia (5). Considering these points, we generated mice transduced with *Selenbp1* in the FC on postnatal day 2 and performed several behavioral analyses of these mice, focusing on PFC-dependent tasks. Mice with FC-transduced *Selenbp1* exhibited deficits in social behaviors similar to hSELENBP1 Tg mice but not in nesting behavior and sucrose preference. These discrepancies may be explained by regional (whole brain vs. FC) and temporal (lifelong vs. developmental period) differences between the two mouse models. For example, the study assessing naturalistic behaviors, anxiety, and cognition of mice with reduced NMDA receptor expression in the whole brain mentioned a possibility that nesting behavior deficits of these mice were indicative of a global impairment (43). Therefore, the reduction of nesting behavior in hSELENBP1 Tg mice might indicate a global impairment rather than a phenotype of schizophrenia.

Previous gene expression analyses of postmortem samples from patients with schizophrenia revealed increased *SELENBP1* expression in the PFC (7). We verified these findings, demonstrating increased SELENBP1 levels in the PFC of patients with schizophrenia compared with those of controls across diverse demographic and postmortem factors, including sex, race, age, PMI, and tissue pH (Fig. 1 and *SI Appendix,* Tables S1 and S2). Consistent with these observations, neuroimaging studies have shown an association between PFC abnormalities and negative symptoms (10–14). The extensive molecular genetic study of schizophrenia implicates glutamatergic dysfunction (44). Interestingly, putative glutamatergic neurons were impaired in the hSELENBP1 Tg mice. Therefore, animal models in the present study may be a translational tool for studying schizophrenia development. Furthermore, given the involvement of various genetic determinants in highly heritable brain disorders, such as schizophrenia and ASD, as demonstrated by genome-wide association studies (45, 46), we examined CNVs and relevant loci in PFC tissues of two patients with schizophrenia with up-regulated *SELENBP1*. These analyses identified overlapping CNVs in lncRNAs of the ASD-related genes, *HERC2,* and *GOLGA8*, in the 15q11.1—11.2 schizophrenia/ASD-associated duplication region (*SI Appendix,* Table S3 for details), suggesting that the pathogenesis of *SELENBP1* upregulation in schizophrenia may be related to that of ASD.

SELENBP1 has been suggested to play a role in the development of schizophrenia; however, the mechanism remains unknown, largely because SELENBP1 is not robustly expressed in the brains of adult humans and rodents (47). Moreover, unlike the vast majority of selenium-related proteins that are abundantly expressed in neurons, SELENBP1 is predominantly expressed in astrocytes (47). Furthermore, in addition to impaired neuronal signaling in the pathophysiology of schizophrenia, several studies have suggested abnormalities in glia and impaired interactions between glia and neurons (48, 49). Hence, neuropathological and behavioral characterization of animals overexpressing cell-type specific SELENBP1 would more clearly define the role of SELENBP1 and provide clues to reveal its mechanism in schizophrenia.

Several studies investigating the development of schizophrenia point to abnormalities in neurodevelopmental processes, such as genetic divergence, exposure to environmental risk factors, inflammation, and an increase in oxidative stress (50–52). Moreover, because physiological sulfide compounds can alleviate oxidative stress, they can provide complementary protection of antioxidant gene expression and storage of sulfides in response to oxidative events (53, 54). Recent genetic and biochemical studies have shown that human SELENBP1 has a 54% similarity to bacterial methanethiol oxidase at the amino acid level (55). Consistent with this, SELENBP1 acts as a methanethiol oxidase that catalyzes the conversion of methanethiol into hydrogen sulfide (H_2_S), formaldehyde, and hydrogen peroxide (H_2_O_2_) in a human fibroblast cell line and mouse erythrocytes (55). In this context, excess hydrogen sulfide and polysulfide production may play a role in the pathogenesis of schizophrenia (51). Thus, it is expected that increased SELENBP1 expression in the brain could produce an excess of sulfur-containing compounds and induce schizophrenia-like abnormalities in behavior and cellular morphology, a possibility that warrants further investigation.

In summary, because human SELENBP1 is a highly conserved protein (56), social-behavioral impairments in both Tg mice carrying the human *SELENBP1* gene and mice transduced with *SELENBP1* into the FC provide clinical and molecular clues to understand the role of SELENBP1 in schizophrenia development. Furthermore, in light of reports that negative symptoms of schizophrenia are a discrete category and are related to dysfunction or hypofunction of dissociable brain circuits, including those in the PFC (18, 57), our results suggest that increased expression of *SELENBP1* in the PFC (BA9) of individuals with schizophrenia is a molecular change relevant to the negative symptoms. The present results also support previous reports documenting the downregulation of SELENBP1 CNVs and proteins in the blood of patients with schizophrenia (2, 3), suggesting that SELENBP1 and its associated biological correlates could potentially be peripheral diagnostic biomarkers of the negative symptoms.

## Materials and Methods

### Collection of Postmortem Human Brain Tissue.

Postmortem human brain tissue (Brodmann area 9), collected during autopsy at the Cuyahoga County Medical Examiner's Office (Cleveland, OH), was supplied by Dr. Stockmeier (Postmortem Brain Core Facility, University of Mississippi Medical Center). Tissue collection and retrospective psychiatric assessments of all subjects were approved by the Institutional Review Board of the University of Mississippi Medical Center (IRB protocol 1999-1002) and University Hospitals Cleveland Medical Center (IRB protocol 11-88-233; see Table 1 for details on the subjects). The Institutional Review Board of Chungnam National University approved the study procedure (IRB No. 201810-BR-168-10).

### Quantitative PCR.

Total RNA from postmortem human PFC tissues was isolated using TRIZOL (Invitrogen). RNA (1 μg) was reverse transcribed into cDNA using oligo d(T) primers and reverse transcriptase (PrimeScript RT-PCR; TAKARA Biomedical Inc.), and 70 ng of the resulting cDNA was used for real-time quantitative PCR (SsoAdvanced Universal SYRB Green Supermix, Bio-Rad). Oligonucleotides targeting SELENBP1 (primer sets #1 to 4; Fig. 1*A* and *SI Appendix,* Table S1) were designed using PRIMEQUEST (Integrated DNA Technologies). The cycle threshold (Ct) values for each target transcript were normalized using the Ct value of glyceraldehyde-3-phosphate dehydrogenase (*GAPDH*) as the reference gene. The relative expression of *SELENBP1* transcripts in patients with schizophrenia was assessed in each pair matched according to sex, race, age, PMI, and tissue pH according to relationship 2^(ΔCt patient – ΔCt healthy)^. Then differences in matched pairs were analyzed by one-sample *t* test using Bio-Rad CFX Manager (Bio-Rad).

### Animals.

Mice were maintained under a 12/12-h light/dark cycle (lights on at 9:00 a.m.), with free access to food and water. Animal care and experimental procedures were approved by the Institutional Animal Care and Use Committee of Chungnam National University guidelines (CNUIACUC No. CNU-01110), INJE University Animal Care and Use Committee (INJE University IACUC No. 2014-53), and Konkuk University’s Council Directive for the Use and Care of Laboratory Animals (KU IACUC No. KU22114).

### Transgene Construction and Generation of hSELENBP1 Tg Mice.

The plasmid, CAGGS-human SELENBP1, was linearized with *Hind*III and *Ssp*I and purified. Linearized plasmid DNA was injected into the male pronucleus of fertilized eggs in C57BL/6J mice. These eggs were transplanted into the oviducts of pseudo-pregnant mice. The Tg founders were bred in wild-type C57BL/6J mice. Animal care and experimental procedures were approved by the Institutional Animal Care and Use Committee of Chungnam National University guidelines (CNUIACUC No. CNU-01110). For details regarding Tg mouse production and protocols, see the *SI Appendix*.

### Lentivirus Construction, Production, and Intracranial Injection into the FC of Neonatal Mice.

Full-length cDNAs for *Selenbp1* (NM_009150.3) and DsRed2 were cloned into the pLenti-M1.4 lentiviral vector backbone containing an IRES-puro^r^ gene cassette under the control of the murine cytomegalovirus (mCMV) immediate-early promoter. Second-generation lentiviral vectors pseudotyped with vesicular stomatitis virus G (VSV-G) were generated by co-transfection of HEK293T cells with the pLenti-M1.4 transfer vector plasmid, psPAX2 plasmid, and VSV-G envelope plasmid using Lipofectamine (Invitrogen, Waltham, MA). Intracranial injections into the FC of neonatal mice were performed as previously described (58). See the *SI Appendix* for further details.

### Detection of SELENBP1.

To verify SELENBP1 expression in Tg and non-Tg mice, as well as normal C57BL/6NTac mice, we performed western blotting, immunohistochemistry, and immunofluorescence staining using mouse anti-SELENBP1 antibodies from OriGene Technologies (TA504700; Rockville, MD) and MBL Inc. (M061-3; Woburn, MA). For details regarding reagents and protocols, see the *SI Appendix*.

### Electrophysiology.

For brain slice used in patch-clamp recordings in the PFC, 4-wk-old SELENBP1 Tg and littermate non-Tg mice were anesthetized with 0.02 mL/g Avertin (2,2,2-tribromoethanol; Sigma-Aldrich, St. Louis, MO). Each mouse brain was quickly removed and coronally sectioned in an artificial cerebrospinal fluid consisting of 130 mM NaCl, 1.25 mM NaH_2_PO_4,_ 3.5 mM KCl, 24 mM NaHCO_3,_ 1.5 mM CaCl_2_-2H_2_O, 1.5 mM MgCl_2_-6H_2_O, and 10 mM glucose. Rostral-to-caudal 300-μm-thick brain slices containing the PFC region were cut using a vibratome (Leica VT1000 S) and bubbled with 95% O_2_/5% CO_2_ (vol/vol) at room temperature. For details regarding recording protocols, see the *SI Appendix*.

### Stereotaxic Surgery and EEG Recordings.

For FC and parietal cortex EEG recordings, age-matched littermates of 5- to 6-mo-old male and female mice underwent the stereotaxic surgery and were given at least 7 d to recover before recording the FC and parietal cortex EEG to auditory stimuli, as reported previously with minor modifications (31). See the *SI Appendix* for details.

### Behavioral Measurements.

A battery of behavioral tests (open-field test, elevated plus-maze test, three-chamber social approach and novelty task, Y-maze spontaneous alternation, nest-building test, forced swim task, sucrose preference task, prepulse inhibition task, and attention set-shift task) was performed using age-matched littermates of 5- to 6-mo-old male mice as reported previously, with minor modifications (59). See the *SI Appendix* for details.

### Statistical Analyses.

All data are expressed as means ± SEM. A one-sample *t* test, paired *t* test, independent *t* test, and ANOVA were used for statistical analyses of all parameters. Group differences were assessed using Sidak’s post hoc test, where necessary. SPSS Statistics 25 (IBM) and Prism 9 software (GraphPad Software) were used for statistical analyses and graphical figures, respectively. The alpha level was set to 0.05.

## Data, Materials, and Software Availability

All study data are included in the article and/or *SI Appendix*.

## Supplementary Material

Appendix 01 (PDF)

## References

[1] M. P. Bansal , DNA sequencing of a mouse liver protein that binds selenium: Implications for selenium’s mechanism of action in cancer prevention. Carcinogenesis **11**, 2071–2073 (1990). 2225343 10.1093/carcin/11.11.2071

[2] S. Amar , Copy number variation of the SELENBP1 gene in schizophrenia. Behav. Brain Funct. **6**, 40 (2010). 20615253 10.1186/1744-9081-6-40 PMC2915948

[3] E. J. Chau , Downregulation of plasma SELENBP1 protein in patients with recent-onset schizophrenia. Prog. Neuropsychopharmacol. Biol. Psychiatry **85**, 1–6 (2018). 29577944 10.1016/j.pnpbp.2018.03.010

[4] S. J. Glatt , Comparative gene expression analysis of blood and brain provides concurrent validation of SELENBP1 up-regulation in schizophrenia. Proc. Natl. Acad. Sci. U.S.A. **102**, 15533–15538 (2005). 16223876 10.1073/pnas.0507666102 PMC1266138

[5] T. Kanazawa , The utility of SELENBP1 gene expression as a biomarker for major psychotic disorders: Replication in schizophrenia and extension to bipolar disorder with psychosis. Am. J. Med. Genet. B Neuropsychiatr. Genet. **147b**, 686–689 (2008). 18163446 10.1002/ajmg.b.30664

[6] T. Kanazawa , Family-based association study of SELENBP1 in schizophrenia. Schizophr. Res. **113**, 268–272 (2009). 19596560 10.1016/j.schres.2009.06.011

[7] M. Udawela , SELENBP1 expression in the prefrontal cortex of subjects with schizophrenia. Transl. Psychiatry **5**, e615 (2015). 26241353 10.1038/tp.2015.108 PMC4564563

[8] N. C. Andreasen, Symptoms, signs, and diagnosis of schizophrenia. Lancet **346**, 477–481 (1995). 7637483 10.1016/s0140-6736(95)91325-4

[9] D. A. Lewis, J. A. Lieberman, Catching up on schizophrenia: Natural history and neurobiology. Neuron **28**, 325–334 (2000). 11144342 10.1016/s0896-6273(00)00111-2

[10] K. Hirao , Theory of mind and frontal lobe pathology in schizophrenia: A voxel-based morphometry study. Schizophr. Res. **105**, 165–174 (2008). 18774263 10.1016/j.schres.2008.07.021

[11] C. I. Hooker, L. Bruce, S. H. Lincoln, M. Fisher, S. Vinogradov, Theory of mind skills are related to gray matter volume in the ventromedial prefrontal cortex in schizophrenia. Biol. Psychiatry **70**, 1169–1178 (2011). 21917239 10.1016/j.biopsych.2011.07.027 PMC3432316

[12] A. Maat, N. E. M. van Haren, C. F. Bartholomeusz, R. S. Kahn, W. Cahn, Emotion recognition and theory of mind are related to gray matter volume of the prefrontal cortex in schizophrenia. Eur. Neuropsychopharmacol. **26**, 255–264 (2016). 26711688 10.1016/j.euroneuro.2015.12.013

[13] C. G. Wible , Prefrontal cortex, negative symptoms, and schizophrenia: An MRI study. Psychiatry Res. **108**, 65–78 (2001). 11738541 10.1016/s0925-4927(01)00109-3 PMC2845854

[14] M. Yamada , Social cognition and frontal lobe pathology in schizophrenia: A voxel-based morphometric study. Neuroimage **35**, 292–298 (2007). 17240165 10.1016/j.neuroimage.2006.10.046

[15] S. G. Shamay-Tsoory, J. Aharon-Peretz, Y. Levkovitz, The neuroanatomical basis of affective mentalizing in schizophrenia: Comparison of patients with schizophrenia and patients with localized prefrontal lesions. Schizophr. Res. **90**, 274–283 (2007). 17182218 10.1016/j.schres.2006.09.020

[16] M. Schneider, M. Koch, Deficient social and play behavior in juvenile and adult rats after neonatal cortical lesion: Effects of chronic pubertal cannabinoid treatment. Neuropsychopharmacology **30**, 944–957 (2005). 15592349 10.1038/sj.npp.1300634

[17] S. W. Kim , Knockdown of phospholipase C-beta1 in the medial prefrontal cortex of male mice impairs working memory among multiple schizophrenia endophenotypes. J. Psychiatry Neurosci. **40**, 78–88 (2015), 10.1503/jpn.130285. 25268789 PMC4354821

[18] B. Kirkpatrick, R. W. Buchanan, The neural basis of the deficit syndrome of schizophrenia. J. Nerv. Ment. Dis. **178**, 545–555 (1990). 2203878 10.1097/00005053-199009000-00001

[19] L. Mei, W. C. Xiong, Neuregulin 1 in neural development, synaptic plasticity and schizophrenia. Nat. Rev. Neurosci. **9**, 437–452 (2008). 18478032 10.1038/nrn2392 PMC2682371

[20] J. K. Millar , Disruption of two novel genes by a translocation co-segregating with schizophrenia. Hum. Mol. Genet. **9**, 1415–1423 (2000). 10814723 10.1093/hmg/9.9.1415

[21] H. Stefansson , Neuregulin 1 and susceptibility to schizophrenia. Am. J. Hum. Genet. **71**, 877–892 (2002). 12145742 10.1086/342734 PMC378543

[22] C. A. Jones, D. J. Watson, K. C. Fone, Animal models of schizophrenia. Br. J. Pharmacol. **164**, 1162–1194 (2011). 21449915 10.1111/j.1476-5381.2011.01386.x PMC3229756

[23] T. Hikida , Dominant-negative DISC1 transgenic mice display schizophrenia-associated phenotypes detected by measures translatable to humans. Proc. Natl. Acad. Sci. U.S.A. **104**, 14501–14506 (2007). 17675407 10.1073/pnas.0704774104 PMC1964873

[24] G. Chana , Biomarker investigations related to pathophysiological pathways in schizophrenia and psychosis. Front. Cell Neurosci. **7**, 95 (2013). 23805071 10.3389/fncel.2013.00095 PMC3693064

[25] H. Y. Koh, D. Kim, J. Lee, S. Lee, H. S. Shin, Deficits in social behavior and sensorimotor gating in mice lacking phospholipase C beta1. Genes. Brain Behav. **7**, 120–128 (2008). 17696993 10.1111/j.1601-183X.2007.00351.x

[26] N. Lijam , Social interaction and sensorimotor gating abnormalities in mice lacking Dvl1. Cell **90**, 895–905 (1997). 9298901 10.1016/s0092-8674(00)80354-2

[27] M. J. Ang, S. Lee, J. C. Kim, S. H. Kim, C. Moon, Behavioral tasks evaluating schizophrenia-like symptoms in animal models: A recent update. Curr. Neuropharmacol. **19**, 641–664 (2021). 32798374 10.2174/1570159X18666200814175114 PMC8573744

[28] R. Nesvag , Regional thinning of the cerebral cortex in schizophrenia: Effects of diagnosis, age and antipsychotic medication. Schizophr. Res. **98**, 16–28 (2008). 17933495 10.1016/j.schres.2007.09.015

[29] P. C. Nopoulos, M. Flaum, N. C. Andreasen, V. W. Swayze, Gray matter heterotopias in schizophrenia. Psychiatry Res. **61**, 11–14 (1995). 7568565 10.1016/0925-4927(95)02573-g

[30] J. Smucny , Evidence for gamma and beta sensory gating deficits as translational endophenotypes for schizophrenia. Psychiatry Res. **214**, 169–174 (2013). 23972946 10.1016/j.pscychresns.2013.07.002 PMC4111638

[31] D. D. Aguilar , Altered neural oscillations and behavior in a genetic mouse model of NMDA receptor hypofunction. Sci. Rep. **11**, 9031 (2021). 33907230 10.1038/s41598-021-88428-9 PMC8079688

[32] A. C. Basu , Targeted disruption of serine racemase affects glutamatergic neurotransmission and behavior. Mol. Psychiatry **14**, 719–727 (2009). 19065142 10.1038/mp.2008.130 PMC2786989

[33] A. Wolkin , Negative symptoms and hypofrontality in chronic schizophrenia. Arch. Gen. Psychiatry **49**, 959–965 (1992). 1360200 10.1001/archpsyc.1992.01820120047007

[34] S. W. Anderson, A. Bechara, H. Damasio, D. Tranel, A. R. Damasio, Impairment of social and moral behavior related to early damage in human prefrontal cortex. Nat. Neurosci. **2**, 1032–1037 (1999). 10526345 10.1038/14833

[35] J. Ko, Neuroanatomical substrates of rodent social behavior: The medial prefrontal cortex and its projection patterns. Front. Neural Circuits **11**, 41 (2017). 28659766 10.3389/fncir.2017.00041 PMC5468389

[36] T. L. Spires , Activity-dependent regulation of synapse and dendritic spine morphology in developing barrel cortex requires phospholipase C-beta1 signalling. Cereb. Cortex **15**, 385–393 (2005). 15749982 10.1093/cercor/bhh141

[37] C. E. McOmish , Phospholipase C-beta1 knockout mice exhibit endophenotypes modeling schizophrenia which are rescued by environmental enrichment and clozapine administration. Mol. Psychiatry **13**, 661–672 (2008). 17667964 10.1038/sj.mp.4002046

[38] S. W. Kim, T. Cho, S. Lee, Phospholipase C-beta1 hypofunction in the pathogenesis of schizophrenia. Front. Psychiatry **6**, 159 (2015). 26635636 10.3389/fpsyt.2015.00159 PMC4648068

[39] N. L. Lazar, N. Rajakumar, D. P. Cain, Injections of NGF into neonatal frontal cortex decrease social interaction as adults: A rat model of schizophrenia. Schizophr. Bull. **34**, 127–136 (2008). 17525084 10.1093/schbul/sbm039 PMC2632378

[40] J. L. Rapoport, J. N. Giedd, N. Gogtay, Neurodevelopmental model of schizophrenia: Update 2012. Mol. Psychiatry **17**, 1228–1238 (2012). 22488257 10.1038/mp.2012.23 PMC3504171

[41] D. R. Weinberger, Implications of normal brain development for the pathogenesis of schizophrenia. Arch. Gen. Psychiatry **44**, 660–669 (1987). 3606332 10.1001/archpsyc.1987.01800190080012

[42] R. C. Saunders, B. S. Kolachana, J. Bachevalier, D. R. Weinberger, Neonatal lesions of the medial temporal lobe disrupt prefrontal cortical regulation of striatal dopamine. Nature **393**, 169–171 (1998). 9603519 10.1038/30245

[43] C. Barkus, L. A. Dawson, T. Sharp, D. M. Bannerman, GluN1 hypomorph mice exhibit wide-ranging behavioral alterations. Genes. Brain Behav. **11**, 342–351 (2012). 22300668 10.1111/j.1601-183X.2012.00767.x PMC3489048

[44] Schizophrenia Working Group of the Psychiatric Genomics, Biological insights from 108 schizophrenia-associated genetic loci. Nature **511**, 421–427 (2014). 25056061 10.1038/nature13595 PMC4112379

[45] C. R. Marshall , Contribution of copy number variants to schizophrenia from a genome-wide study of 41,321 subjects. Nat. Genet. **49**, 27–35 (2017). 27869829 10.1038/ng.3725 PMC5737772

[46] D. Pinto , Functional impact of global rare copy number variation in autism spectrum disorders. Nature **466**, 368–372 (2010). 20531469 10.1038/nature09146 PMC3021798

[47] A. R. Sasuclark, V. S. Khadka, M. W. Pitts, Cell-type specific analysis of selenium-related genes in brain. Antioxidants **8**, 120 (2019). 31060314 10.3390/antiox8050120 PMC6562762

[48] H. G. Bernstein, J. Steiner, P. C. Guest, H. Dobrowolny, B. Bogerts, Glial cells as key players in schizophrenia pathology: Recent insights and concepts of therapy. Schizophr. Res. **161**, 4–18 (2015). 24948484 10.1016/j.schres.2014.03.035

[49] D. Kondziella , Glial-neuronal interactions are impaired in the schizophrenia model of repeated MK801 exposure. Neuropsychopharmacology **31**, 1880–1887 (2006). 16395297 10.1038/sj.npp.1300993

[50] C. W. Holtzman , Stress and neurodevelopmental processes in the emergence of psychosis. Neuroscience **249**, 172–191 (2013), 10.1016/j.neuroscience.2012.12.017. 23298853 PMC4140178

[51] M. Ide , Excess hydrogen sulfide and polysulfides production underlies a schizophrenia pathophysiology. EMBO Mol. Med. **11**, e10695 (2019). 31657521 10.15252/emmm.201910695 PMC6895609

[52] L. M. Jansen, C. C. Gispen-de Wied, R. S. Kahn, Selective impairments in the stress response in schizophrenic patients. Psychopharmacology **149**, 319–325 (2000). 10823414 10.1007/s002130000381

[53] W. N. Niu, P. K. Yadav, J. Adamec, R. Banerjee, S-glutathionylation enhances human cystathionine beta-synthase activity under oxidative stress conditions. Antioxid. Redox Signal. **22**, 350–361 (2015). 24893130 10.1089/ars.2014.5891 PMC4307034

[54] J. L. Wallace, R. Wang, Hydrogen sulfide-based therapeutics: Exploiting a unique but ubiquitous gasotransmitter. Nat. Rev. Drug. Discov. **14**, 329–345 (2015). 25849904 10.1038/nrd4433

[55] A. Pol , Mutations in SELENBP1, encoding a novel human methanethiol oxidase, cause extraoral halitosis. Nat. Genet. **50**, 120–129 (2018). 29255262 10.1038/s41588-017-0006-7 PMC5742538

[56] P. W. Chang , Isolation, characterization, and chromosomal mapping of a novel cDNA clone encoding human selenium binding protein. J. Cell Biochem. **64**, 217–224 (1997). 9027582 10.1002/(sici)1097-4644(199702)64:2<217::aid-jcb5>3.0.co;2-#

[57] J. J. Shaffer , Neural correlates of schizophrenia negative symptoms: Distinct subtypes impact dissociable brain circuits. Mol. Neuropsychiatry **1**, 191–200 (2015). 27606313 10.1159/000440979 PMC4996000

[58] N. Pilpel, N. Landeck, M. Klugmann, P. H. Seeburg, M. K. Schwarz, Rapid, reproducible transduction of select forebrain regions by targeted recombinant virus injection into the neonatal mouse brain. J. Neurosci. Methods **182**, 55–63 (2009). 19505498 10.1016/j.jneumeth.2009.05.020

[59] N. Zhang , S-SCAM, a rare copy number variation gene, induces schizophrenia-related endophenotypes in transgenic mouse model. J. Neurosci. **35**, 1892–1904 (2015). 25653350 10.1523/JNEUROSCI.3658-14.2015 PMC4315826

