# Supplementary material for: SELENBP1 overexpression in the prefrontal cortex underlies negative symptoms of schizophrenia
Source: Proc Natl Acad Sci U S A. 2022 Dec 13;119(51):e2203711119. doi: 10.1073/pnas.2203711119 (PMC9907074; doi:10.1073/pnas.2203711119)
Supplement: Supplementary file 1 — Appendix 01 (PDF) [file pnas.2203711119.sapp.pdf]

SUPPORTING INFORMATION (SI)

for

" SELENBP1 overexpression in the prefrontal cortex underlies negative symptoms of schizophrenia "

Soojin Kim *et. al.*

SUPPLEMENTARY MATERIALS & METHOD

Collection of postmortem human brain tissues

Postmortem human brain tissue (Brodmann area 9) was supplied by Craig A. Stockmeier, Ph.D., Post-mortem Brain Core Facility of University of Mississippi Medical Center. Tissues were collected at autopsy at the Cuyahoga County Medical Examiner's Office, Cleveland, OH, USA. Tissue collection and retrospective psychiatric assessments of all subjects were carried out as described in (1) and approved by the Institutional Review Board of the University of Mississippi Medical Center (IRB protocol 1999-1002) and University Hospitals Cleveland Medical Center (IRB protocol. 11-88-233, see Table 1 for details on the subjects). Furthermore, the Institutional Review Board of Chungnam National University approved the study procedure (IRB No. 201810-BR-168-10).

RNA extraction from the human brain tissues and RT-qPCR assay

Total RNA from postmortem human prefrontal cortex (PFC) tissues was isolated using TRIZOL (Invitrogen, Waltham, MA, USA) to verify alterations in *SELENBP1* expression in the brains of schizophrenia patients. One microgram of RNA was reverse transcribed using oligo d(T) primer and reverse transcriptase (PrimeScript^{TM} RT-qPCR, TAKARA Biomedical Inc., Japan). Oligonucleotides were designed to target SELENBP1 (primer set #1-4, Fig. 1A and Table S1) using PRIMEQUEST (Integrated DNA Technologies, Coralville, IA, USA). cDNA (70 *ng*) was used for real-time quantitative polymerase chain reaction (RT-qPCR). The threshold cycle (Ct) values for each target transcript were normalized to that of the gene encoding glyceraldehyde 3-phosphate dehydrogenase (GAPDH) by calculating ΔCt. Relative expression of *SELENBP1* transcripts in schizophrenia patients was assessed by $2^{(\Delta Ct\ patient - \Delta Ct\ healthy)}$ in each pair matched with

gender, race, age, postmortem interval (PMI), and tissue pH using the Bio-Rad CFX Manager (Bio-Rad, Hercules, CA, USA) (Fig. 1B and Table 1). Then, we analyzed the differences in matched pairs using a one-sample t-test.

Transgene construction and generation of SELENBP1 Tg mice

CAGGS-human *SELENBP1*, a 6,203 bp recombinant plasmid vector for expression of the human *SELENBP1*, was constructed by inserting human *SELENBP1* cDNA under a robust synthetic promoter system consisting of a hybrid of CMV enhancer/chicken β-actin (CBA) promoter, the first exon/intron of the CBA gene, and the splice acceptor of the rabbit beta-globin gene (commonly called the CAGGS promoter) (2) (Fig. S1A). The plasmid CAGGS-human *SELENBP1* was linearized with *Hind*III and *Ssp*I and purified. The linearized plasmid DNA was injected into the male pronucleus of fertilized eggs in C57BL/6J mice. These eggs were then transplanted into the oviducts of pseudo-pregnant mice. Tg founders were bred with wild-type C57BL/6J mice. Mice were maintained under a 12/12-h light/dark cycle (lights on at 9:00 a.m.), with free access to food and water. Animal care and experimental procedures were approved by the Institutional Animal Care and Use Committee of the Chungnam National University guidelines (CNUIACUC No. CNU-01110).

Genotyping of Tg mice by PCR

The primers were designed using the CAGGS promoter and human *SELENBP1*-coding sequences. Oligonucleotides for targeting h*SELENBP1* Tg transgene (Table S1) were designed and amplified for genotyping. The PCR procedures were as follows: beginning 5 min at 95°C, 35 cycles of 94°C denature for 30 sec, annealing at 62°C for 30 sec, and extension at 72°C for 1 min.

The following primers were used: a chimeric intron-forward primer (F): 5’-AAC CAT GTT CAT GCC TTC TTC T-3'; and a human *SELENBP1*-reverse primer (R): 5’-GGT TGA AGC CAT CTC GTA AGA C-3', resulting in a 733 bp band (Fig. 2B). Rod cGMP-specific 3',5'-cyclic phosphodiesterase subunit beta (*Pde6b*) gene was used as an internal control in PCR, resulting in a 240 bp band. The PCR conditions were as follows: 5 min at 95 °C, 35 cycles of denaturation at 94 °C for 30 s, annealing at 62 °C for 30 s, and extension at 72 °C for 1 min.

Western blotting

Mice were anesthetized with an isoflurane inhalant and perfused with cold phosphate-buffered saline (PBS). The tissues were placed in a grinding tube (CKMix50R, Bertin Co., France) containing beads, PBS, and protease inhibitor cocktail (p3100-001, GenDEPOT, Katy, TX USA) for 15 s to make the homogenate using a homogenizer (Precellys 24, Bertin Co.). Tissue lysates were prepared using radioimmunoprecipitation assay (RIPA) buffer containing a protease inhibitor cocktail. Protein concentrations were quantified using a bicinchoninic acid assay (23227, Thermo Scientific, Waltham, MA, USA). Two or twenty micrograms of total protein from each sample were loaded in duplicate onto 10 % Mini-PROTEAN TGX Precast Gels (456-8034, Bio-Rad) and separated by electrophoresis (120 V constant), then transferred (100 V constant) for 1 h 30 min onto polyvinylidene difluoride (PVDF) membranes. The membranes were then blocked in 5% non-fat milk powder/Tris-buffered saline with 0.1% Tween-20 (TBST) for 1 h at room temperature. After being washed in TBST buffer, the membrane was incubated with mouse anti-SELENBP1 antibody (1:1000, TA504700, OriGene Technologies, Rockville, MD, USA) overnight at 4 °C, followed by incubation with anti-mouse secondary antibody (1:1000, Bio-Rad) for 1 h at room temperature. Protein levels were represented as chemiluminescence

signals after incubation with Clarity Max Western ECL substrate (1705062, Bio-Rad) and detected using a ChemiDoc MP Imaging System (Bio-Rad) (Fig. S1C). Band intensity was quantified using Image Lab 6.0 software.

Immunohistochemistry and immunostaining

To verify SELENBP1 expression in the PFC and hippocampus of Tg and non-Tg mice, we performed histological analysis as described previously (3). Briefly, the mice were anesthetized and transcardially perfused with 4 % paraformaldehyde in PBS. The brains were post-fixed with the same solution, after which sagittal sections were prepared. Brain sections were stained using incubation with mouse anti-SELENBP1 primary antibody (1:500; TA504700, OriGene Technologies) and then with horseradish peroxidase (HRP)-conjugated secondary antibody (1:500; Abcam, Cambridge, England). Brain sections were visualized with the HRP substrate 3, 3′-diaminobenzidine (DAB) in 0.1 M Tris buffer. Sections were mounted in Vectashield mounting media with or without diamidino-2-phenylindole (DAPI; Vector Laboratories, Burlingame, CA, USA).

Frozen brain sections were washed with PBS to remove antifreeze medium, followed by permeabilization in 0.5% Triton-X 100 in Tris-buffered saline for 20 min and blocking with 5% goat serum, 5% horse serum, and 5% donkey serum plus 3% bovine serum albumin in PBS for 2 h at room temperature. The frozen sections were then incubated with primary antibodies. The sections were washed with PBS and incubated for 1 h at room temperature with secondary antibodies conjugated fluorescence. Primary antibodies used were anti-NeuN antibody (1:500, MAB377, Millipore, Burlington, MA, USA), anti-GFAP antibody (1:500, MA512023, Thermo Scientific), or anti-SELENBP1 antibody (1:500, M061-3, MBL Inc, Woburn, MA, USA).

Images were captured and analyzed using a confocal laser scanning microscope (TCS Sp8, Leica, Wetzlar, Germany) and microscopic software (LAS X Core, Leica).

Nissl staining

Cresyl violet stock solution (0.01 % cresyl violet-acetate) and buffer solution {0.094 M acetic acid and 0.01 M sodium acetate (pH 3.5) were prepared. The cresyl violet working solution and jar were incubated for at least an hour at 60°C before staining. The rehydrated sectioned tissues were incubated in pre-warmed cresyl violet working solution at 60°C for 8 - 14 min. The stained tissues were under dehydration. The stained tissues were mounted using Xylene-based mounting media and coverslips.

Electrophysiology

For brain slice patch-clamp recordings in the PFC (Fig. 5), four-week-old SELENBP1 Tg and littermate non-Tg mice were anesthetized with 0.02 ml/g Avertin (2,2,2-Tribromoethanol, Sigma-Aldrich, St. Louis, MO, USA). Each mouse brain was quickly removed and coronally sectioned in artificial cerebrospinal fluid (ACSF) consisting of 130 mM NaCl, 1.25 mM NaH_2PO_4, 3.5 mM KCl, 24 mM $NaHCO_3$, 1.5 mM $CaCl_2$-$2H_2O$, 1.5 mM $MgCl_2$-$6H_2O$, and 10 mM glucose. Rostral-to-caudal 300-μm-thick brain slices containing the PFC region were cut using a vibratome (Leica VT1000 S) bubbled with 95% O_2/5% CO_2 (vol/vol) at room temperature. Intrinsic firing properties of neurons in coronal sections containing the PFC with ACSF. Recording electrodes were pulled from borosilicate glass capillaries (1B150F-4, World Precision Instruments, Sarasota, FL, USA) and had a tip resistance of 3-7 MΩ when filled with an intracellular solution consisting (mM) of 140 K-gluconate, 10 HEPES, 7 NaCl, 4 Mg-ATP, and 0.3 Na-GTP (280-290 mOsmol / l, pH 7.4). Signals were amplified using a MultiClamp 700A amplifier (Axon Instruments, Berkeley,

CA, USA) and digitalized using Digidata 1322A (Axon Instruments). Recordings used Clampex 11.1 software and were considered acceptable only when the seal resistance was greater than 1 GΩ, and the access resistance was less than 10-30 MΩ. A patched cell was inspected in current-clamp mode. In current-clamp mode, neurons were injected with 10 depolarizing step current pulses in 20 pA increments for 1 s each at 3.5 s intervals, after which the membrane potentials were initially adjusted to -60 mV by injecting current ranging from -100 to -20 pA. Our criteria for putative neurons is a capacitance range from 20 to 80 pF and a half-time range from 1 ms to 15 ms for each non-fast action potential (4).

Lentivirus production

Viral constructs. The full-length cDNAs of *Selenbp1* (NM_009150.3) and DsRed2, red fluorescent protein, were amplified by performing PCR (Table S1). Lentiviral vector encoding *Selenbp1* was constructed by inserting the amplified cDNA of *Selenbp1* into a pLenti-M1.4, lentiviral vector backbone containing an IRES-puro^r gene cassette under the control of the murine cytomegalovirus (mCMV) immediate-early promoter (Fig. 4B). The cDNA of DsRed2 substituted the cDNA of *Selenbp1* to construct a lentiviral control vector (Fig. S4B-1).

Viral packaging. Vesicular stomatitis virus G (VSV-G) pseudo-typed lentiviral vectors were generated by co-transfection of lentiviral transfer vector plasmid, gag-pol plasmid, rev plasmid, and VSV-G envelope plasmid into HEK293T cells using Lipofectamine (Invitrogen). At 48 h after transfection, the supernatant was filtered using a 0.45 µm CE syringe and concentrated with an Amicon Ultra 100 centrifugation device (Millipore). These concentrated lentiviral vectors were purified using ultracentrifugation. To determine the titer of viral vectors, HeLa cells were cultured in a 5×10^4/well plate filled with 1 ml Dulbecco's Modified Eagle's medium supplemented with 10 % fetal bovine serum. At

24 h after cell culture, the number of cells in each well was counted. The concentrated viral vectors were 10-fold diluted with polybrene (Final Conc., 8 μg/ml), and diluted viral vectors replaced the culture medium of HeLa cells. At 48 h after transduction, puromycin (final Conc., 1 μg/ml) was added for 4 days. The transduced HeLa cells were cultivated for 1 week until the cells formed visible colonies. The colonies were stained with crystal violet, and the number of colonies was counted. *In vitro* biological activity was confirmed by transduction of HCT116 cells which do not express Selenbp1, followed by western blot analyses for confirming SELENBP1 expression.

Intracranial injections into the frontal cortex (FC) of neonatal mice

Intracranial injections into the FC of neonatal mice were performed as previously described (5, 6). Neonatal male wild-type mice (postnatal day 2) in a C57BL/6J mouse (Jackson Laboratory, Bar Harbor, ME, USA) were cryo-anesthetized at 0 °C for 3 min before injection. Following cessation of movement, 0.5 μL of high-titer recombinant mouse Selenbp1 or DsRed2 lentiviral vectors (1×10^9 TU/ml) were bilaterally injected into the frontal cortex areas (-1.0 anterior-posterior, ± 0.5 mediolateral, -0.7 dorsoventral) by referring to a neonatal brain atlas (6), at a rate of 0.1 μL/min using a 10 μL Hamilton syringe (Hamilton, Reno, NV, USA) equipped with a beveled 34-gauge needle. After the injections, the injected neonatal mice were transferred to a foster mother for care. Animal care and experimental procedures followed the guidelines of the INJE University Animal Care and Use Committee (INJE University IACUC No. 2014-53).

RNA extraction from the brain tissues of LV-injected mice and RT-qPCR assay

To verify the expression levels of ectopic and endogenous mouse *Selenbp1* transcripts in the FC areas of LV-injected mice, we collected FC samples from LV-mCMV-

Selenbp1- and LV-mCMV-*DsRed2*-injected mice. The concentration of total RNA was determined by measuring the absorbance at 260 nm (A260) using a NanoDrop spectrophotometer (NanoDrop™ 2000 Spectrophotometer, Thermo Scientific™). Total RNA (1 µg) was treated with 122.5 U DNase I (Invitrogen) in a 100 µL reaction for 1 h at 37 °C. The enzyme was denatured at 90 °C for 10 min, and 2.5 µL of the solution was added to each reaction tube. RT-qPCR was performed using One-Step SYBR PrimeScript RT-qPCR kit Ver. 1 (RR066A, Takara) and a Bio-Rad CFX96 Touch Real-Time PCR Detection System (Bio-Rad) according to the manufacturer's instructions and cycling conditions of 42 °C for 5 min, 95 °C for 10 s, followed by 40 cycles of 95 °C for 5 s and 62 °C for 30 s. One hundred *nanograms* of RNA were used as a template, and the reactions were performed in triplicate at 20 µL volume. The primers were designed to amplify the 5' UTR region of lentiviral ectopic *Selenbp1* and endogenous *Selenbp1* (NM_009150.3) (see Table S1). *Gus,* mouse *β*-glucuronidase mRNA, was used as an internal control because the level of *Gus* mRNA does not change during the development of the whole mouse brain and spinal cord (7). One hundred nanograms of cDNA were used for the RT-qPCR assay. Relative quantification of the *Selenbp1* transcript was normalized to the Ct value of *Gus* internal control, and relative fold expression of ectopic *Selenbp1* transcript in LV-mCMV-*Selenbp1*-injected mice was compared with LV-mCMV-DsRed2 injected mice using RT-qPCR and the $2^{-\Delta\Delta Ct}$ method (8).

Stereotaxic surgery and EEG recording

Age-matched littermates of 5- to 6-month-old male and female mice were deeply anesthetized with isoflurane (4% induction, 1–2% maintenance) and body temperature was maintained with a chemical heating pad throughout the surgery. Epidural EEG screw electrodes (0.10″, Cat No. 8403, Pinnacle Technology Inc., Lawrence, Kansas, USA) were implanted in the skull above the frontal cortex (from bregma: A/P+1.9 mm, M/L −1.0 mm)

and parietal cortex (A/P −1.6 mm, M/L+1.7 mm) with a reference and ground screw implanted above the olfactory bulb (from lambda, A/P −4.9 mm, M/L±0.8 mm). Animals were given at least 7 days to recover from surgery before any experiments began. While individual mice were placed in a holding Plexiglass cage, EEG signals were amplified using a Digital Lynx SX (Neuralynx, Montana, USA), filtered (0.5-210 Hz, and digitized at 2000 Hz. Following a 10-min tethered habituation period, auditory stimuli were presented as pairs of 80 dB 5 kHz tones of 50 ms duration (n = 100 trials) with an inter-trial interval (ITI) of 6 s and a 500 ms interstimulus interval (ISI).

The average event-related potential (ERP) for the first (S1) and second (S2) stimuli were analyzed as described in the earlier study (9). Briefly, a waveform average of 100 paired-tone presentations was created from raw EEG records and baseline-corrected to the average signals during the 100-ms period before each stimulus onset. Then, we measured the maximum positive defection around 20 ms (P20, 15–30 ms after each tone onset) and the maximum negative defection around 40 ms (N40, 25–55 ms). In addition, we also analyzed time-frequency power spectra around the two paired stimuli within the frequency range of 1 to 100 Hz, using multi-taper spectral estimation implemented in the Chronux toolbox (Cold Spring Harbor, New York, USA) with a 250-ms sliding window stepped by 15 ms and a time-bandwidth product of 3 with 5 tapers. Evoked spectral powers were normalized by dividing each data point by the average power during a 1.5-s pre-stimulus period (-2 to -0.5 s) at the corresponding frequency. Average beta and gamma powers during the presentation of each tone (50-ms duration) were measured in 15-25 Hz and 26-50 Hz ranges, respectively.

Behavioral measurements

All behavioral tests were performed with age-matched littermates of 5- to 6-month-old male and female mice, as reported previously with minor modifications (10).

Open-field test. A behavioral assay for locomotor activity and anxiety was performed according to previously described procedures (3). Each mouse was placed in the central square region (20 cm × 20 cm) of an open-field box (45 cm × 45 cm × 45 cm). The extent of their spontaneous movement over the course of 30 min and time spent in the central sector of the open field during a 10 min observation period were analyzed using Smart 3.0 software (Panlab Harvard Apparatus, Holliston, MA, USA). During the analysis, the open field was divided into 16 small cubic areas using the software. The central sector of the open field was defined as four small cubic areas in the central area.

Elevated-plus maze test. Behavioral assays for anxiety were performed as previously described (3). Each mouse was placed in an elevated (70 cm above floor level) plus maze with two opposite open arms (28 cm × 5 cm each) and two opposite closed arms (28 cm × 5 cm each) with 15 cm-high walls, and the number of entries into individual arms and the time spent on individual arms were measured for 7 min.

Three-chamber social approach and novelty tasks A behavioral task for the social approach and social novelty was performed as previously described (11). The three-chamber task consisted of three sessions. In the first session, each mouse was placed in a three-chambered apparatus with two small containers in the left or right (not center) chamber and allowed to freely explore each chamber for 10 min for habituation. After 10 min, the mouse was guided to the center chamber, and the two entrances to the center chamber were blocked, while a novel inanimate object (Object) and an unfamiliar Stranger mouse (Stranger 1) were placed in the two containers. Then, the two entrances were opened to allow the mouse to freely explore the new environment for 10 min. In the third session, the test mouse was guided to the center chamber again, with a blockade of the entrances. The object was replaced with a new Stranger mouse (Stranger 2), followed by an exploration of the now-familiar Stranger 1 or novel unfamiliar Stranger 2 by the test mouse for 10 min. The amount

of time spent exploring an inanimate object, or a Stranger mouse, was analyzed by observing the exploration behaviors of the test mouse, such as approaching, sniffing, and direct contact using the nose or forelimb. Exploration of the Object or the Stranger was defined as the length of time showing that the test mouse (*i*) orients its nose towards and comes close to the Object or the Stranger, 1 cm away from the container with the Object or the Stranger; or (*ii*) touches the container with the Object or the Stranger using nose or whisker; or (*iii*) grabs the container with its forelimbs while hindlimbs are fixed. Blind analysis was performed on mouse behavior. In addition to time exploring an object or a Stranger mouse, we used the preference scores, which were calculated as the ratio of the numerical difference between times spent exploring the targets (Stranger 1 vs. Object, or Stranger 2 vs. now-familiar Stranger 1) divided by the total time spent exploring both targets, as described previously (11, 12). Positive scores indicated a preference for the novel social stimulus relative to the non-social or familiar social stimulus, whereas negative scores reflected a preference for the non-social or familiar stimulus, and scores approximating "0" indicated no preference.

Y-maze spontaneous alternation. A behavioral assay for working memory was performed as described previously (11). The Y-maze apparatus, made of Plexiglas, had three equally spaced arms (120°, 37 cm × 7.5 cm × 12.5 cm). Specific motifs were decorated on the walls of each arm, thus allowing visual discrimination. Each mouse was placed at the end of one arm and allowed to freely explore the apparatus for 7 min. The arms were labeled A, B, or C, and the sequence of arm entries was manually recorded. Entry was defined as all body and tail into the arm. Spontaneous alternation was defined as successive entries into each of the three arms on an overlapping triplet set (for example, ABC, BCA, CAB). The percentage of spontaneous alternation performance was defined as the ratio of actual alternation (total alternations) to possible alternations (total arm entries -2) × 100.

Nest-building test. Mice were housed individually and provided with cotton nestled square (5 cm × 5 cm; Ancare) as a nest-building material 1 h before the onset of the dark phase. Nests were assessed the next morning, and the quality of the nest was scored on a 5-point rating scale, as described previously (10, 13).

Sucrose preference task. Behavioral assays for anhedonia were carried out as previously reported, with minor modifications (10, 14). Before the experiment, mice were singly housed and trained to drink sugary water (2% sucrose solution) for 2 days. Mice were then water-deprived for 18 h and then exposed to one bottle of 2% sucrose and one bottle of water for 1 h. Then, the bottles were placed at opposite ends of the home cage. The total consumption of each fluid was measured, and sucrose preference was defined as the average sucrose consumption ratio during the choice period. The sucrose consumption ratio was calculated by dividing the total consumption of sucrose by the total consumption of both water and sucrose.

Forced Swim Task. Mice were placed into a bucket (20 cm high and 15 cm in diameter filled 15 cm high) filled with 24 °C tap water. Behavior was videotaped from above for 6 minutes, with the last four minutes scored by a blinded experimenter for time climbing, swimming, and immobile. An increase in immobility or decrease in active behavior reflects a depression-like phenotype.

Prepulse Inhibition. Mice were placed into startle chambers that consisted of a Plexiglas cylinder resting on a Plexiglas platform in a sound-attenuating, ventilated chamber (San Diego Instruments, CA). Sessions were 30 minutes long. The first 10 minutes consisted of acclimation to the background (70 dB) noise. The testing consisted of a pseudo-random representation of eight different types of trials: a 40-millisecond broadband 74, 82, 90, 120 dB burst (pulse alone trial); three different prepulse pulse trials in which 20-millisecond long 74 dB, 82 dB, or 90 dB preceded the 120 dB pulse by 100 milliseconds. The intertrial interval

was 15 sec. Prepulse inhibition (PPI) ratio was calculated as [(startle amplitude $_{pulse}$ – startle amplitude $_{prepulse + pulse}$)/startle amplitude $_{pulse}$) × 100].

Attentional set-shifting task (ASST) The experiments were performed according to the procedure with some modifications for mice, described in the previous studies (15, 16). During the experiment, mice were singly housed and food-restricted to maintain them at 90% of their *ad libitum* weight.

Apparatus. All behavior tests took place in an opaque acrylic box (50 × 37.5 × 25 cm) with a transparent front side. The box was equally divided into two compartments by a black sliding door, which created a starting area and a test area. Two ceramic pots (7 cm diameter, 3.5 cm height) were located in the test area in order to create the odorant and texture medium. Two pots were filled with a texture medium, and 50 µl of odorant was placed on top of the medium. The reward was placed at the bottom of the ceramic pot and concealed by a medium. The food reward was 1/8 piece of Kellogg's® Froot Loops cereal.

Habituation and shaping. One day prior to testing, each mouse was habituated to the apparatus, pots, and food reward for 5 min. Then, mice were permitted to dig freely in the pots. The reward was put on the medium of two pots at the start of shaping, and then the consecutive baited reward was placed increasingly deeper in the bowl. During the shaping session, home cage bedding was used for medium. If mice found the reward in both pots, the trial was restarted. After 5 min, a new trial was restarted even if mice had not found both rewards. Trials continued until the mice dug the pots for six trials continuously to find a hidden reward in the bottom of the pots, or they were stopped after 60 min. If mice failed to reliably dig the pot after 60 min, they were trained the next day again.

Test. Following initial shaping, each mouse was trained to discriminate between pairs of pots that differed in odor and/or the media covering the food reward (Table S4). This

discrimination training was performed during two sessions, which consisted of several phases and trials. During each trial, the mouse was placed in the starting area, and the sliding door was raised to give the mouse access to two posts, only one of which contained the food reward. The first four trials were discovery trials. In discovery trials, even after an incorrect choice, the mouse was permitted to dig and collect food rewards from the other pot, but errors were recorded. After the discovery trials, if the mouse started digging in the no-reward pot, the trial was immediately terminated, and the mouse was returned to the starting area. These trials were recorded as errors. Trials continued until the mouse reached a criterion of six consecutive correct trials. In the first session, the mice performed simple discrimination (SD) consisting of two different media, followed by compound discrimination (CD), during which a novel odor was added as an irrelevant stimulus. Then, the mouse performed CD reversal (CDrev), during which the CD stimuli were presented again, but the former positive exemplar was switched to being the negative relevant stimulus. In the subsequent intra-dimensional shift (IDS) and IDS reversal (IDS rev.), the stimuli were changed completely. A new stimulus was introduced, but the dimension of relevant and irrelevant stimuli was the same as in prior phases. The next day, in the second session, the mice additionally performed IDS2 and IDS2rev. And then, extra-dimensional shift (EDS), the final phase of the test, was conducted. In the EDS, new stimuli were presented, but the relevant dimension was switched. The formerly relevant dimension (medium) became the irrelevant dimension, and the formerly irrelevant dimension (odor) became relevant.

CGH array

Comparative genomic hybridization microarray. Two brain samples (SCZ-A and SCZ-C) with schizophrenia, which show higher expression levels of SELENBP1 (1.7 and 3-fold, respectively) than the healthy samples, were selected. DNA of the two samples was

extracted from dlPFC. After extracting the DNA, we labeled 50~500 ng of both test and reference DNA with Cy 3- and Cy 5-dCTP. For the WGS reference, hg19 was selected, and NA10851 was chosen as the reference for aCGH. The platform used for this experiment was SurePrein G3 Human CGH 2x400K microarray (Algilent Inc. Santa Clara, CA, USA). Standard Agilent procedures were performed using the Agilent G4900DA SureScan Microarray Scanner. The extracted data was processed using genomic workbench v11.0.1.1 to visualize, detect and analyze aberration patterns from CGH microarray profiles.

Analysis of CNVs in the *SELENBP1*-overexpressed PFC of two schizophrenia patients

In the present study, Tg mice carrying human *SELENBP1* and overexpressed FC-specific *Selenbp1* exhibited social impairments but no other schizophrenic-like endophenotypes, such as hyperlocomotion and deficits of working memory. These results led us to speculate that the upregulation of SELENBP1 might be related to other mental disorders with asociality, including autism spectrum disorder (ASD). Although schizophrenia and ASD are clinically distinct disorders, studies have suggested that genetic overlap is present (17, 18). Notably, genetic and chromosomal studies, including CNVs analysis, have characterized disease-relevant genes and loci related to schizophrenia and ASD (17, 18).

Therefore, we performed array comparative genomic hybridization (aCGH) to analyze CNVs of the two *SELENBP1*-overexpressed postmortem brains of schizophrenia patients (SCZ-A and SCZ-C, Fig. 1B and Tables S1 and S2) and two healthy matched subjects (Healthy-A and Healthy-C, Table 1). We found that CNVs of the *SELENBP1*-overexpressed PFC tissues of subjects with schizophrenia partially overlapped with the CNVs of long non-coding RNAs (lncRNAs) of ASD-related genes such as the HECT domain, RLD2 (*HERC2*), and Golgin A8 family member C (*GOLGA8*) in the schizophrenia- and ASD-relevant 15q11.1-11.2 duplication region (19-22) (Table S3).

ASD risk genes include protein-coding genes commonly influenced by CNVs, which can perturb gene expression. However, reported ASD risk genes comprise only a small proportion of ASD cases (23, 24). ASD pathogenesis is involved in diverse genetic risk factors, including regulatory lncRNAs, defined as transcripts greater than 200 nucleotides in length that do not encode proteins (25). LncRNAs are potentially key regulators of neurodevelopmental functions such as brain development, neural differentiation, and synaptic plasticity (26-28), and contribute to cognitive function and neuronal tissue specification (29). Moreover, because lncRNAs are tissue-specific and highly expressed in the human brain, they are likely involved in complex neurodevelopmental disorders, such as ASD (26, 29-32). Previously, CNVs of both *HERC2* and lncRNAs of *HERC2* have been found in the 15q11-13 duplication region in ASD pathogenesis (33). CNVs of lncRNAs of *HERC2*, *HERC2P3*, and *HERC2P8* in the 15q11.1-11.2 region were found in the *SELENBP1*-overexpressed brains of schizophrenia patients (Table S3). For example, a homozygous missense mutation of *HERC2* in humans causes developmental delay in neuropsychiatric diseases, including ASD (34, 35).

These results suggest that *SELENBP1* upregulation in the brain might be involved in the pathological processes related to ASD with social deficits, as well as in the development of schizophrenia.

SUPPLEMENTARY FIGURES & TABLES

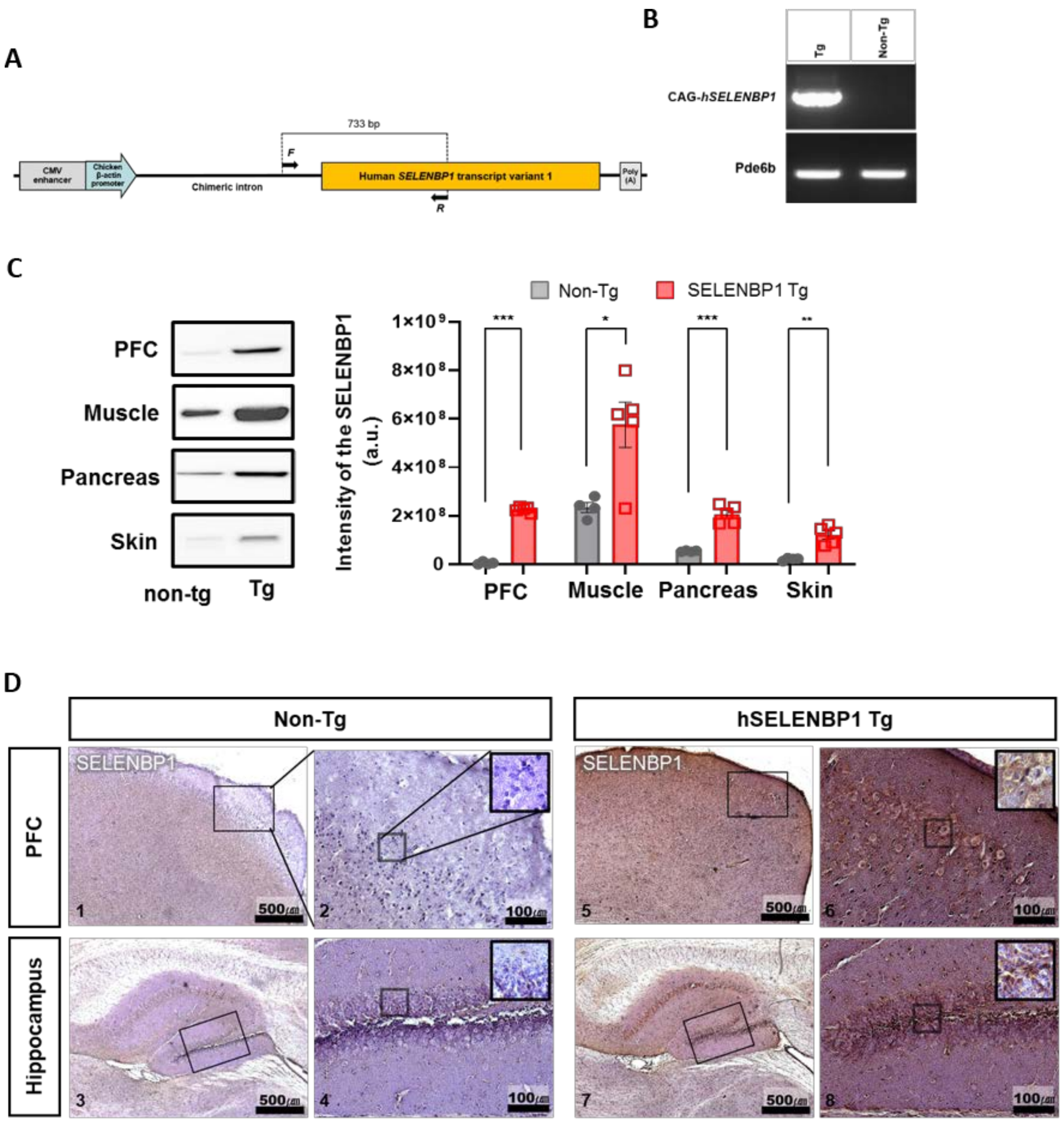

Fig. S1. Verification of hSELENBP1 Tg mice. (*A, B*) Schematic representation of the human *SELENBP1* transgene constructs and 733-bp PCR products. (*C*) Representative Western blot images showing SELENBP1 expression in the PFC, muscle, pancreas, and skin of non-Tg control and hSELENBP1 Tg mice. SELENBP1 levels, expressed in arbitrary units (a.u.), were normalized using total proteins and Bio-Rad image lab software (right panel). Data are expressed as means ± SEM (Non-Tg, n = 4; Tg, n = 5; $*p < 0.05$, $**p < 0.01$, $***p < 0.001$). (*D*) Immunohistochemical staining for hSELENBP1 in the PFC and hippocampus of non-Tg control and hSELENBP1 Tg mice (5-8). The rectangles in 1, 3, 5, and 7 indicate subregions of 2, 4, 6, and 8, respectively. Scale bars: 500 µm (1, 3, 5, and 7) and 100 µm (2, 4, 6, and 8).

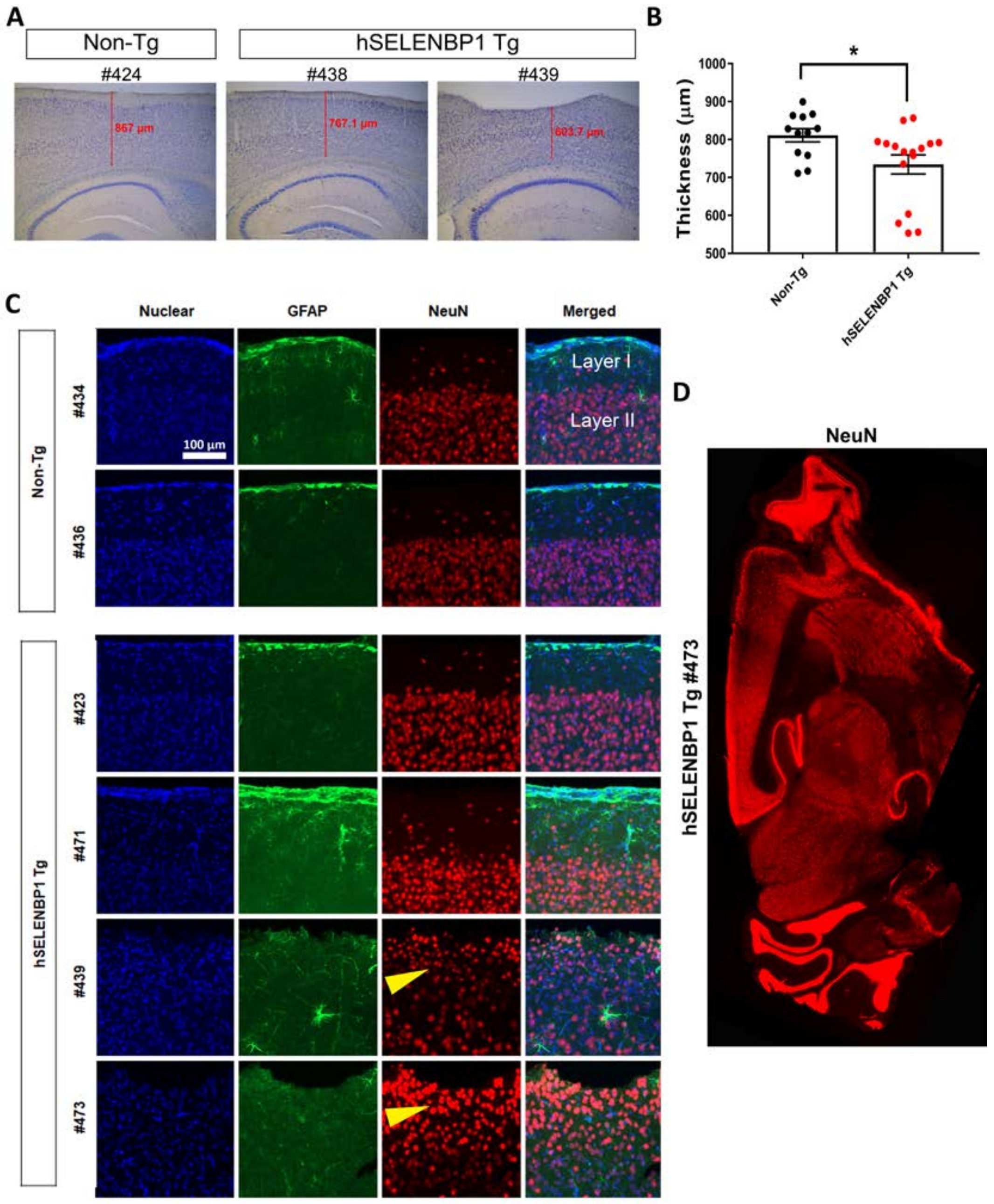

Fig. S2. Neuroanatomical characterization of hSELENBP1 Tg mice. (*A*) Representative images (50x) of Nissl-stained hSELENBP1 Tg and non-Tg mouse cortices. (*B*) The cortex of hSELENBP1 Tg mice was thinner than that of non-Tg mice. Data are expressed as means ± SEM (n = 4 sections/mouse from 3-4 mice/group). (*C*) Cortical ectopias in the hSELENBP1 Tg mouse. NeuN immunostaining revealed heterotopias and ectopias in upper-layer cortical neurons of hSELENBP1 Tg mice (yellow arrowhead regions). (*D*) Overall view of a NeuN-immunostained sagittal section from a Tg mouse.

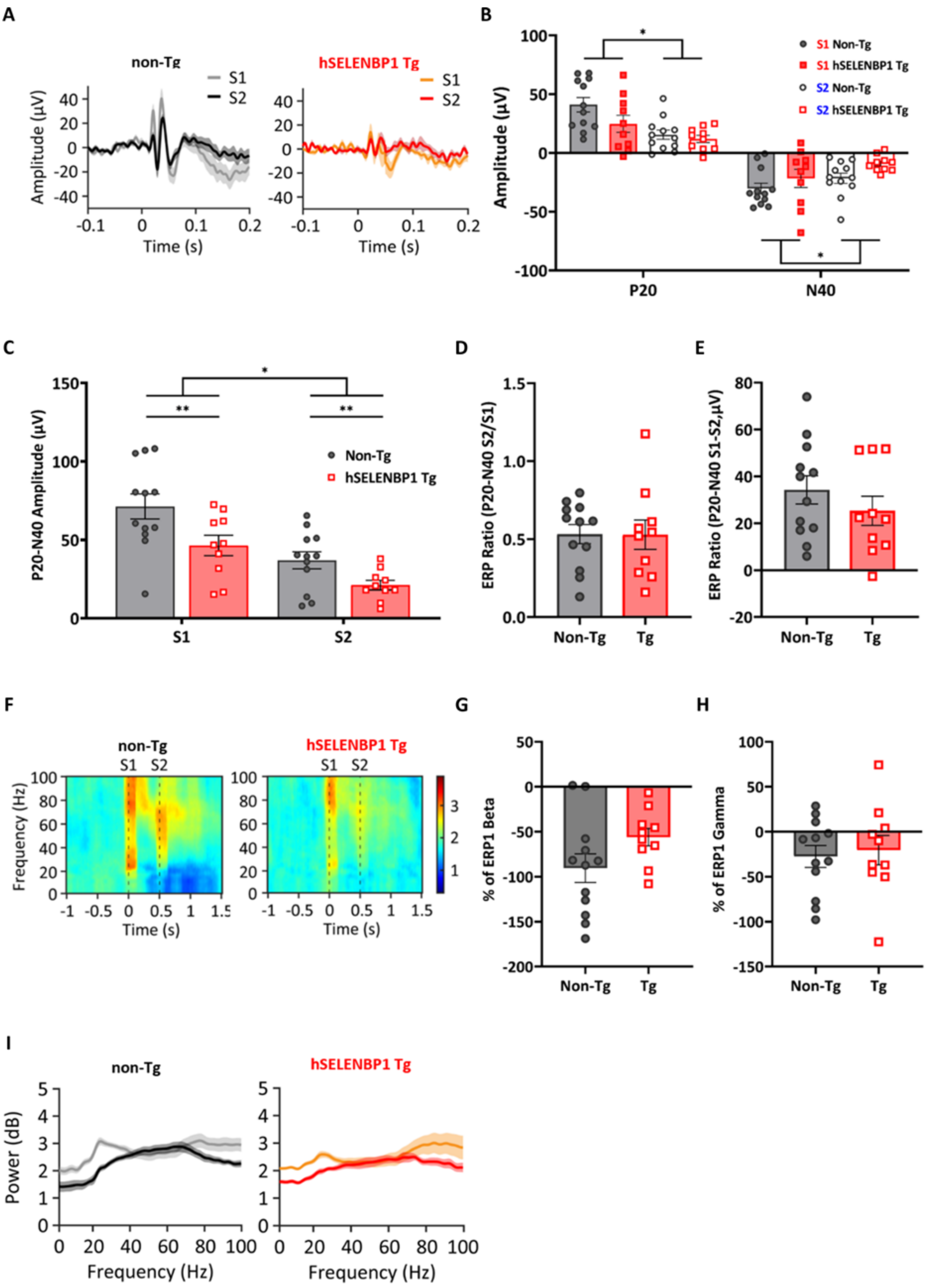
A
non-Tg
S1
S2
Amplitude (µV)
Time (s)
hSELENBP1 Tg
B
Amplitude (µV)
S1 Non-Tg
S1 hSELENBP1 Tg
S2 Non-Tg
S2 hSELENBP1 Tg
P20
N40
C
P20-N40 Amplitude (µV)
Non-Tg
hSELENBP1 Tg
S1
S2
D
ERP Ratio (P20-N40 S2/S1)
Non-Tg
Tg
E
ERP Ratio (P20-N40 S1-S2,µV)
F
Frequency (Hz)
Time (s)
G
% of ERP1 Beta
H
% of ERP1 Gamma
I
Power (dB)
Frequency (Hz)

Fig. S3. Parietal cortex ERP in the non-Tg and hSELENBP1 mice. (*A*) The parietal cortex EEGs of the non-Tg (n = 12) and hSELENBP1 (n = 10) mice were measured by averaging the evoked related potential (ERP) of 100 repetitions of two identical 5kHz 50 ms tones (S1 and S2) with 50 ms interstimulus interval. (*B)* Both non-Tg mice and hSELENBP1 Tg mice exhibited significantly larger P20 and N40 in S1 than S2, but no between-group difference in P20 (two-way RM ANOVA, Group, $F_{1,20}$ = 2.46, p = 0.13; P20, $F_{1,20}$ = 19.89.57, p < 0.001; Interaction, $F_{1,20}$ = 2.22, p = 0.15) and N40 ($F_{1,20}$ = 3.27, p = 0.09; N40, $F_{1,20}$ = 6.67, p < 0.05; Interaction, $F_{1,20}$ = 0.22, p = 0.64). (*C)* Both non-Tg and hSELENBP1 Tg mice had significant reduction in the P20-N40 amplitudes from S1 to S2 and between-group difference was found (two-way RM ANOVA, Group; $F_{1,20}$ = 7.08, p < 0.05; P20-N40, $F_{1,20}$ = 46.58, p < 0.001; Interaction, $F_{1,20}$ = 1.06, p = 0.32). (*D, E)* No between-group differences in the normalized ratios S2/S1 (t_{20} = -0.38, *p* = 0.97) and S1-S2 (t_{20} = 1.03, *p* = 0.32) were observed. (*F)* Power spectrum density in response to two auditory stimuli (S1 [0-0.05 s] and S2 [0.5-0.55 s]). (*G)* Both non-Tg and hSELENBP1 mice displayed comparable reductions from S1 to S2 in the beta (15-25 Hz) power (t_{20} = -1.76, *p* = 0.09) and gamma (26-50 Hz) power (t_{20} = -0.35, *p* = 0.73). *(I)* There was no significant between-group difference (t_{20} = 0.88, *p* = 0.39) in S1 (ERP1) across the entire frequency range (2-100 Hz), but significant group difference (t_{20} = 2.13, *p* = 0.05) in S2 (ERP2). Data are presented as means ± SEM.

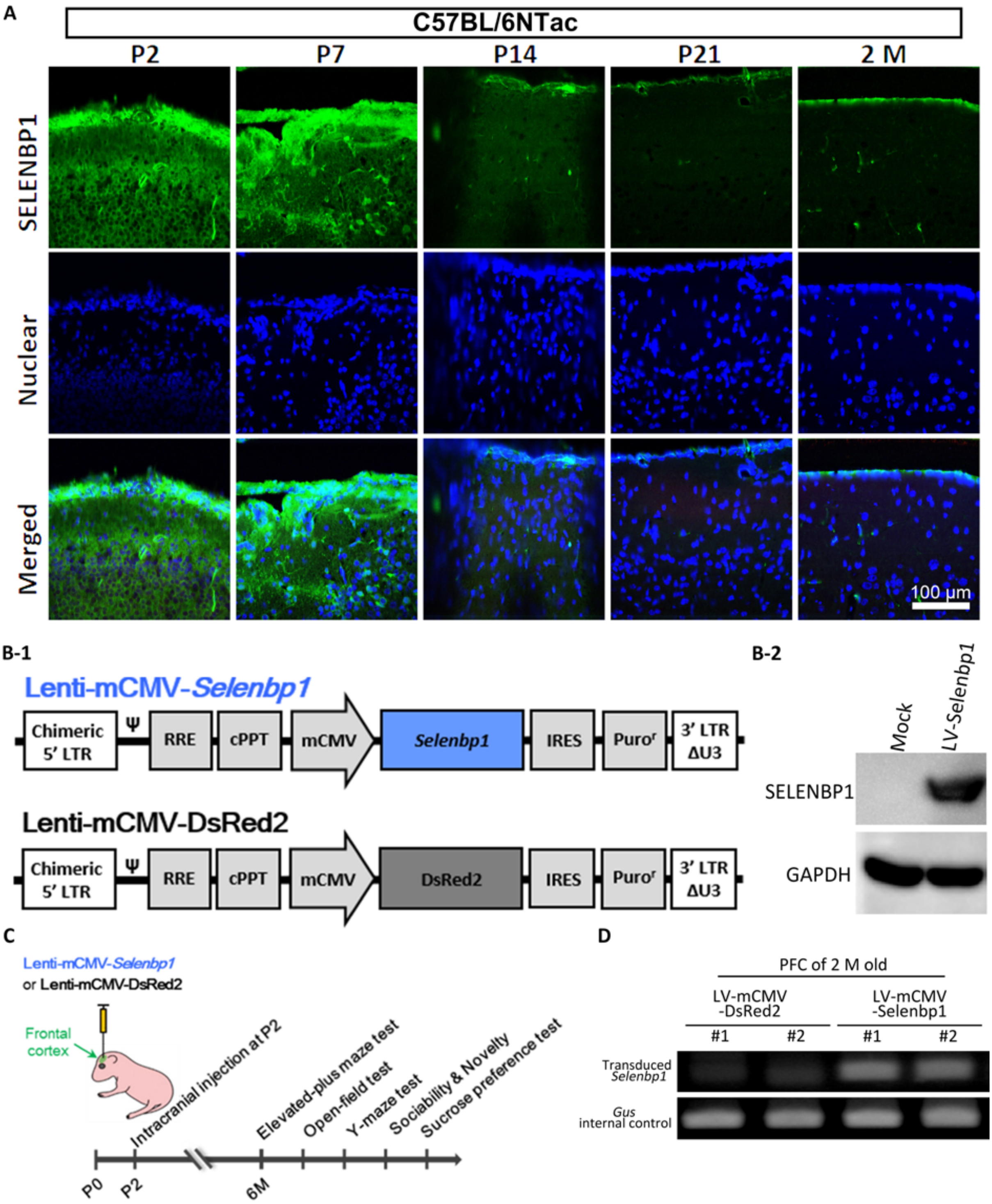

Fig. S4. Lentivirus-mediated overexpression of *Selenbp1* in the mouse FC. (*A*) Time-dependent decrease in SELENBP1 in the cortex of a non-transduced C57B/6 mouse brain. (*B*) Construction and delivery of *Selenbp1* lentivirus (LV) vectors. (*B-1*) Constructs of LV vectors encoding *Selenbp1* cDNA (upper) and *DsRed2* cDNA (lower). (*B-2*) Western blot of mouse SELENBP1 expressed in human colon cancer cell line HCT116, which does not express *SELENBP1*. (*C*) Schematic depicting the temporal strategies for LV injection and behavioral tests. The LV vectors were administered into the FC of neonatal wild-type mice. P2, postnatal day 2; 6M, 6-months-old. (*D*) Expression level of transduced *Selenbp1* in the FC of 2-month-old mice injected with LV-mCMV-*Selenbp1*, measured by

RT-qPCR assay. The primer set targeting the 5' UTR of the *Selenbp1* transgene was used (see Table S1). Internal control, mouse β-glucuronidase (Gus); UTR, untranslated region.

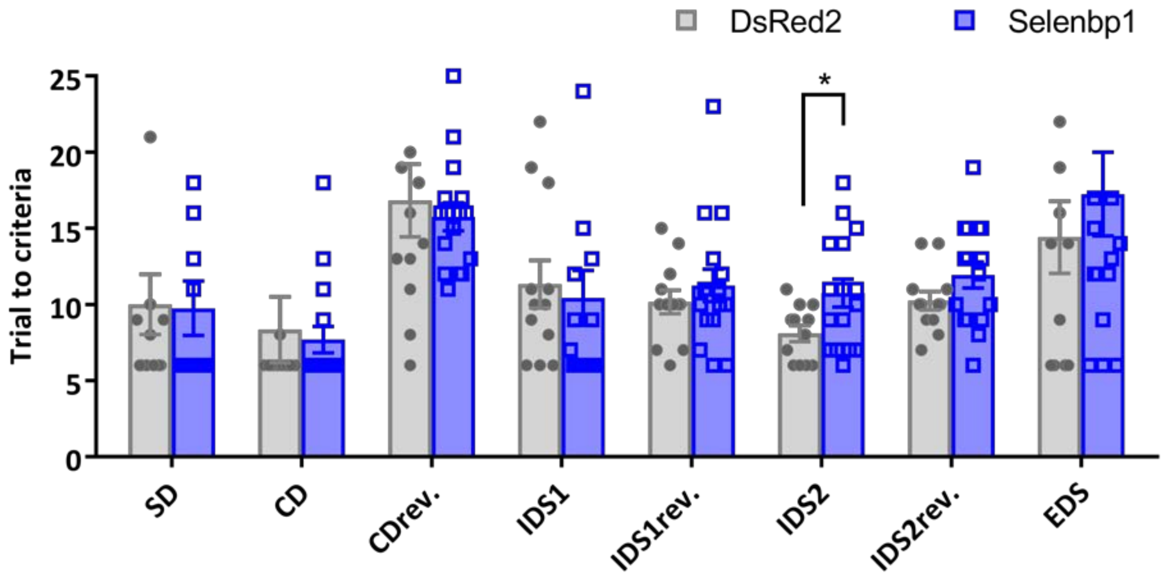

Fig. S5. The number of trials to reach the criterion in each session of the attentional set-shifting task.

Mice with FC-transduced Selenbp1 (n = 16) and *DsRed2* control (n = 12) mice showed comparable performances in the SD, CD, CD, IDS1, IDS1 reversal, IDS2 reversal, and EDS session. However, mice with FC-transduced Selenbp1 took significantly more trials to reach the criterion in the IDS2 session than *DsRed2* control mice (*). SD, simple discrimination; CD, compound discrimination; CD Rev, compound discrimination reversal; IDS, intra-dimensional shift; IDS rev, intra-dimensional shift reversal; IDS2 rev, intra-dimensional shift 2 reversal; EDS, extra-dimensional shift.

Table S1. Primer list

Target	Primer	Oligonucleotide sequence (5’ -> 3’)	PCR product (bp)
SELENBP1 3’UTR	#1-F	CACTGTTGCTTGTTGCTCAC	81 bp
	#1-R	GAAGGACAGGGTTACGAGTTTAT	
SELENBP1 transcript variant 1 only	#2-F	AGCATGGCTACGAAATGTGGGA	190 bp
	#2-R	GGTGGATGACCTGGCAATAC	
SELENBP1 transcript variant 1 and 3	#3-F	GTCTACCTGCCCTGCATTTA	105 bp
	#3-R	GTGGATGACCTGGCAATACT	
SELENBP1 exon 7	#4-F	ACTGTACGGGAGCCACTTAT	98 bp
	#4-R	AGCGGATCTCCAAGGGAATA	
SELENBP1 Tg	Chimeric intron-F	AAC CAT GTT CAT GCC TTC TTC T	733 bp
	hSELENBP1-R	GGT TGA AGC CAT CTC GTA AGA C	
Pde6b internal control	Pde6b WT	ACCTGCATGTGAACCCAGTATTCTATC	240 bp
	Pde6b Common	CTACAGCCCCTCTCCAAGGTTTATAG	
Selenbp1 CDS	XbaI-Selenbp1-F	ACT CTA GAA CTA GTG GAT CCA TGG CTA CAA AAT GCA CAA AGT	1443 bp
	ClaI-Selenbp1-R	TTA TCG ATT CAG ATC CAG ATG TCA GAA CTG CAG	
DsRed2	BamHI-Kozak-DsRed2-F	GAT CCC GCC ACC ATG GCC TCC TCC GAG AAC GTC AT	702 bp
	ClaI-DsRed2-R	GGT ATC GAT TTA CAG GAA CAG GTG GTG GCG GCC CT	
Transduced *Selenbp1*	LV-Selenbp1-F	CAC CGT AGA ACG CAG AAC TCT A	149 bp
	LV-Selenbp1-R	GTG CCT GTG TTC CGG TAA AT	
Endogenous *Selenbp1*	mSelenbp1-F	CCC AAC TTT CTG GTG GAC TTT	188 bp
	mSelenbp1-R	TCA GAG TGG CCT TGG AGA GTT G	
Gus internal control	mGus-F	CCG ACC TCT CGA ACA ACC G	169 bp
	mGus-R	GCT TCC CGT TCA TAC CAC ACC	

Table S2. The expression level of SELENBP1 transcripts in the postmortem human BA9 samples.

Primer set	Subject	Mean efficiency corrected Ct	Normalized expression	Relative expression (fold change)	Compared to regulation threshold	P-value
#1 (3' UTR)	SCZ-A	25.06	0.03103	1.7902	Up regulated	0.00254
	Healthy-A	25.03	0.01734	1	No change	N/A
	SCZ-B	26.13	0.0089	1.67103	Up regulated	0.001781
	Healthy-B	28.22	0.00533	1	No change	N/A
	SCZ-C	25.75	0.01911	1.64442	Up regulated	0.005018
	Healthy-C	25.77	0.01162	1	No change	N/A
	SCZ-D	24.85	0.02015	1.60024	Up regulated	0.003875
	Healthy-D	26.97	0.01259	1	No change	N/A
	SCZ-E	25.82	0.03513	1.56918	Up regulated	0.006217
	Healthy-E	27.34	0.02238	1	No change	N/A
	SCZ-F	23.31	0.01035	0.37311	Down regulated	0.000395
	Healthy-F	24.14	0.02775	1	No change	N/A
#2 (Exon 2-4)	SCZ-A	26.34	0.02089	2.53663	Up regulated	0.002242
	Healthy-A	26.82	0.00823	1	No change	N/A
	SCZ-B	27.24	0.00569	1.00083	No change	0.987525
	Healthy-B	28.56	0.00568	1	No change	N/A
	SCZ-C	26.43	0.01738	2.59669	Up regulated	0.000035
	Healthy-C	27.15	0.00669	1	No change	N/A
	SCZ-D	25.54	0.01437	1.17257	No change	0.078846
	Healthy-D	27.31	0.01226	1	No change	N/A
	SCZ-E	27.64	0.01543	0.77283	No change	0.054246
	Healthy-E	28.09	0.01997	1	No change	N/A
	SCZ-F	25.94	0.00691	0.32884	Down regulated	0.000056
	Healthy-F	26.43	0.02101	1	No change	N/A
#3 (Exon 3-4)	SCZ-A	29.98	0.00322	2.00379	Up regulated	0.000578
	Healthy-A	30.45	0.00161	1	No change	N/A
	SCZ-B	30.71	0.00117	1.17715	No change	0.029529
	Healthy-B	32.23	0.001	1	No change	N/A
	SCZ-C	30.21	0.00279	2.40242	Up regulated	0.000073
	Healthy-C	30.84	0.00116	1	No change	N/A
	SCZ-D	29.41	0.00311	1.53092	Up regulated	0.000862
	Healthy-D	31.28	0.00203	1	No change	N/A
	SCZ-E	30.43	0.00363	1.18917	No change	0.048348
	Healthy-E	31.45	0.00305	1	No change	N/A
	SCZ-F	29.51	0.00159	0.49693	Down regulated	0.001038
	Healthy-F	30.43	0.00321	1	No change	N/A
#4 (Exon 7)	SCZ-A	25.3	0.02065	2.03302	Up regulated	0.002322
	Healthy-A	25.58	0.01016	1	No change	N/A
	SCZ-B	25.05	0.00588	0.9649	No change	0.570791
	Healthy-B	26.82	0.00609	1	No change	N/A
	SCZ-C	24.25	0.01828	2.16952	Up regulated	0.000017
	Healthy-C	24.83	0.00843	1	No change	N/A
	SCZ-D	23.65	0.01962	1.30972	No change	0.002379
	Healthy-D	25.64	0.01498	1	No change	N/A
	SCZ-E	26.08	0.02023	1.20795	No change	0.013538
	Healthy-E	27.04	0.01675	1	No change	N/A
	SCZ-F	26.11	0.00752	0.36548	Down regulated	0.000039
	Healthy-F	26.37	0.02057	1	No change	N/A

SCZ, Patients with schizophrenia; Healthy, Non-psychiatric control subjects; UTR, Untranslated Region; Ct, Cycle quantification value.

Table S3. CNVs in the SELENBP1-overexpressed postmortem BA9 tissues of patients with schizophrenia.

**Sample**	**Cytogenetic band**	**Genomic position (bp)**	**Size (kb)**	**CNV**	**Genes involved**	**Relevance**
SCZ-A	1q21.1	144,986,366 - 145,076,206	89.840	Gain	*BC065231, BX647792, LOC100288142, NBPF9, LOC653513, PDE4DIP*	Events in SCZ
SCZ-C	1q21.1	144,986,366 - 145,076,206	89.840	Gain	*BC065231, BX647792, LOC100288142, NBPF9, LOC653513, PDE4DIP*	Events in SCZ
SCZ-A	1q44	248,738,898 - 248,798,161	59.263	Loss	*OR2T10, OR2T11*	
SCZ-C	1q44	248,738,898 - 248,798,161	59.263	Loss	*OR2T10, OR2T11*	
SCZ-A	2p11.2	89,135,619 - 90,038,017	902.398	Gain	*AK128525, IGKV, IGVK-A2, Ig kappa, abParts*	
SCZ-C	2p11.2	89,135,619 - 90,265,119	1129.500	Gain	*AK128525, IGKV, IGVK-A2, Ig kappa, abParts*	
SCZ-C	2p11.2	89,151,282 - 89,160,192	8.910	Gain	*AK128525, lg kappa, abParts*	
SCZ-A	2p11.2	90,226,406 - 90,265,119	38.713	Gain	*abParts*	
SCZ-A	2p22.3	34,697,718 - 34,730,142	32.424	Loss	*Mir_548*	
SCZ-C	2p22.3	34,697,718 - 34,730,142	32.424	Gain	*Mir_548*	
SCZ-A	3q26.1	162,514,534 - 162,619,141	104.607	Gain	*BC073807*	
SCZ-C	3q26.1	162,514,534 - 162,619,141	104.607	Gain	*BC073807*	
SCZ-A	4q13.2	69,392,545 - 69,483,277	90.732	Gain	*UGT2B17*	
SCZ-C	4q13.2	69,392,545 - 69,483,277	90.732	Gain	*UGT2B17*	
SCZ-A	4q32.3	168,810,790 - 168,992,014	181.224	Gain	*ANXA10*	
SCZ-C	4q32.3	168,810,790 - 168,992,014	181.224	Gain	*ANXA10*	
SCZ-A	4q35.2	190,488,733 - 190,678,708	189.975	Gain	*BC087857*	Events in ACRD
SCZ-C	4q35.2	190,488,733 - 190,678,708	189.975	Gain	*BC087857*	Events in ACRD
SCZ-A	5q31.3	140,223,256 - 140,236,399	13.143	Gain	*PCDHA1, PCDHA10, PCDHA2, PCDHA3, PCDHA4, PCDHA5, PCDHA6, PCDHA7, PCDHA8, PCDHA9*	
SCZ-C	5q31.3	140,223,256 - 140,236,399	13.143	Gain	*PCDHA1, PCDHA10, PCDHA2, PCDHA3, PCDHA4, PCDHA5, PCDHA6, PCDHA7, PCDHA8, PCDHA9*	

SCZ-A	5q35.3	180,418,512 - 180,426,430	7.918	Loss	*BTNL3*	
SCZ-C	5q35.3	180,418,512 - 180,429,788	11.276	Gain	*BTNL3*	
SCZ-A	6p22.1	29,854,870 - 29,902,314	47.444	Gain	*AK097625, BC035647, HCG4B, HLA-G, HLA-H, HLA-J*	
SCZ-C	6p22.1	29,854,870 - 29,902,314	47.444	Gain	*AK097625, BC035647, HCG4B, HLA-G, HLA-H, HLA-J*	
SCZ-A	6p21.32	32,450,699 - 32,630,524	179.825	Gain	*AK293020, HLA-DQA1, HLA-DQB1, HLA-DRB1, HLA-DRB5, HLA-DRB6*	
SCZ-C	6p21.32	32,501,547 - 32,518,700	17.153	Loss	*HLA-DRB1, HLA-DRB5*	
SCZ-A	7q33	133,788,914 - 133,797,387	8.473	Gain	*LRGUK*	
SCZ-C	7q33	133,788,914 - 133,797,387	8.473	Gain	*LRGUK*	
SCZ-A	10q11.22	46,968,072 - 47,695,889	727.817	Loss	*AGAP9, AK057316, AK309109, ANXA8, BMS1P6, DQ588224, FAM25C, FAM35DP, GPRIN2, HNRNPA1P33, LINC00842, NPY4R, SYT15*	
SCZ-C	10q11.22	46,968,072 - 47,702,587	734.515	Loss	*AGAP9, AK057316, AK309109, ANXA8, BMS1P6, DQ588224, FAM25C, FAM35DP, GPRIN2, HNRNPA1P33, LINC00842, NPY4R, SYT15*	
SCZ-A	10q21.1	56,448,627 - 56,468,820	20.193	Loss	*PCDH15*	Events in SCZ
SCZ-C	10q21.1	56,448,627 - 56,468,820	20.193	Loss	*PCDH15*	Events in SCZ
SCZ-A	11p15.4	5,792,682 - 5,805,665	12.983	Gain	*OR52N5*, TRIM22, TRIM5*	Events in ACRD
SCZ-C	11p15.4	5,785,900 - 5,805,665	19.765	Gain	*OR52N5*, TRIM22, TRIM5*	Events in ACRD
SCZ-A	11q11	55,368,154 - 55,430,765	62.611	Loss	*OR4C11,OR4P4,OR4S2*	
SCZ-A	12p13.2	11,218,244 - 11,249,210	30.966	Loss	*PRB4, PRH1-PRR4, TAS2R43*	

SCZ-C	12p13.2	11,218,244 - 11,239,663	21.419	Loss	*PRB4, PRH1-PRR4*	
SCZ-A	13q21.1	57,760,478 - 57,787,399	26.921	Loss	*PRR20E*	
SCZ-C	13q21.1	57,760,478 - 57,787,399	26.921	Loss	*PRR20E*	
SCZ-A	14q32.33	106,243,209 - 106,810,907	567.698	Gain	*ADAM6, AK128652, BC042994, DKFZp686O16217, FLJ00382, IGH@, IGHD, IGHE, IGHG1, KIAA0125, LINC00226, Z49973, abParts*	
SCZ-C	14q32.33	10,6263,088 - 106,775,018	511.930	Gain	*ADAM6, AK128652, BC042994, DKFZp686O16217, FLJ00382, IGH@, IGHD, IGHE, IGHG1, KIAA0125, LINC00226, abParts*	
SCZ-A	14q32.33	107,148,739 - 107,180,095	31.356	Gain	*abParts*	
SCZ-C	14q32.33	107,148,739 - 107,180,095	31.356	Gain	*abPartsC*	
SCZ-A	15q11.1 -11.2	20,432,851- 22,578,630	2145.779	Gain	*AJ004954, CHEK2P2, CT60, CXADRP2, DQ571479, DQ572979, DQ573684, DQ576041, DQ578838, DQ582073, DQ582260, DQ582939, DQ583164, DQ587539, DQ590589, DQ592463, DQ594309, DQ595048, DQ595648, DQ600342, DQ786202, GOLGA6L6, GOLGA8CP*, HERC2P3*, HERC2P7*, JB175342, LOC646214, LOC727924, NBEAP1, NF1P2,OR4M2, OR4N3P, OR4N4, POTEB, REREP3, abParts*	Events in SCZ, Autism-related 15q11-13 duplication region
SCZ-C	15q11.2	20,849,110 - 21,933,378	1084.268	Loss	*CT60, DQ571479, DQ573684, DQ576041, DQ595048, LOC646214, NBEAP1, NF1P2, POTEB*	Events in SCZ, Events in ACRD
SCZ-A	20p13	1,563,715 - 1,584,485	20.700	Loss	*SIRPB1*	

SCZ-C	20p13	1,563,715 - 1,577,359	13.644	Loss	*SIRPB1*	
SCZ-A	22q11.22	22,455,436 - 23,245,888	790.452	Gain	*BCR, DKFZp667J0810, DQ570150, DQ575049, DQ597441, GGTLC2, IGLL5, LOC648691, LOC96610,MIR650, POM121L1P, PRAME, VPREB1, ZNF280A, ZNF280B, abParts*	Events in ACRD
SCZ-C	22q11.22	22,455,436 - 23,245,888	790.452	Gain	*BCR, DKFZp667J0810, DQ570150, DQ575049, DQ597441, GGTLC2, IGLL5, LOC648691, LOC96610, MIR650, POM121L1P, PRAME, VPREB1, ZNF280A, ZNF280B, abParts*	Events in ACRD
SCZ-A	22q11.23	24,347,959 - 24,395,353	47.394	Gain	*GSTT1, GSTTP2, LOC391322*	Events in SCZ
SCZ-C	22q11.23	24,347,959 - 24,395,353	47.394	Gain	*GSTT1, GSTTP2, LOC391322*	Events in SCZ

SCZ, Subjects with schizophrenia; CNVs, copy number variations; ACRD, Autism Chromosome Rearrangement Database; *, Genes overlapped with the Autism database (www.mindspec.org/autdb.html).

Table S4. Detailed illustration of the ASST procedure

Phase	Dimension	Discrimination 1		Discrimination 2	
Session 1					
SD	Media	Plastic bead vs. C*at litter*			
CD	Media	Benzaldehyde<br>Plastic bead	Geraniol<br>*Cat litter*	Benzaldehyde<br>*Cat litter*	Geraniol<br>Plastic bead
CDrev	Media	Benzaldehyde<br>*Plastic bead*	Geraniol<br>Cat litter	Benzaldehyde<br>Cat litter	Geraniol<br>*Plastic bead*
IDS	Media	Limonen<br>Corncob	Butanol<br>*Wood pellet*	Limonen<br>*Wood pellet*	Butanol<br>Corncob
IDSrev	Media	Limonen<br>*Corncob*	Butanol<br>Wood pellet	Limonen<br>Wood pellet	Butanol<br>*Corncob*
Session 2					
IDS2	Media	Decanal<br>*Styrofoam ball*	Acetic acid<br>Wood shavings	Decanal<br>Wood shavings	Acetic acid<br>*Styrofoam ball*
IDS2rev	Media	Decanal<br>Styrofoam ball	Acetic acid<br>*Wood shavings*	Decanal<br>*Wood shavings*	Acetic acid<br>Styrofoam ball
EDS	Odors	Propylacetate<br>Dry cellulose	*Hexanal*<br>Sawdust	Propylacetate<br>Sawdust	*Hexanal*<br>Dry cellulose

Mice were presented with either discrimination 1 or 2. Correct reward-relevant stimulus is indicated in *italics*.

References

1. H. Zhu *et al.*, Quantitative analysis of focused a-to-I RNA editing sites by ultra-high-throughput sequencing in psychiatric disorders. *PLoS One* **7**, e43227 (2012).
2. S. J. Gray, Optimizing promoters for recombinant adeno-associated virus-mediated gene expression in the peripheral and central nervous system using self-complementary vectors. *Human Gene Therapy, 22:1143–1153* 10.1089/hum.2010.245 (2011).
3. S. W. Kim *et al.*, Knockdown of phospholipase C-beta1 in the medial prefrontal cortex of male mice impairs working memory among multiple schizophrenia endophenotypes. *J Psychiatry Neurosci., 40(2):78-88.* 10.1503/jpn.130285 (2015).
4. L. G. Nowak, R. Azouz, M. V. Sanchez-Vives, C. M. Gray, D. A. McCormick, Electrophysiological classes of cat primary visual cortical neurons in vivo as revealed by quantitative analyses. *J Neurophysiol* **89**, 1541-1566 (2003).
5. J. Y. Kim *et al.*, Viral transduction of the neonatal brain delivers controllable genetic mosaicism for visualising and manipulating neuronal circuits in vivo. *Eur J Neurosci* **37**, 1203-1220 (2013).
6. N. Pilpel, N. Landeck, M. Klugmann, P. H. Seeburg, M. K. Schwarz, Rapid, reproducible transduction of select forebrain regions by targeted recombinant virus injection into the neonatal mouse brain. *J Neurosci Methods* **182**, 55-63 (2009).
7. D. Xu *et al.*, Identifying suitable reference genes for developing and injured mouse CNS tissues. *Dev Neurobiol* **78**, 39-50 (2018).
8. K. J. Livak, T. D. Schmittgen, Analysis of relative gene expression data using real-time quantitative PCR and the 2(-Delta Delta C(T)) Method. *Methods* **25**, 402-408 (2001).
9. D. D. Aguilar *et al.*, Altered neural oscillations and behavior in a genetic mouse model of NMDA receptor hypofunction. *Sci Rep* **11**, 9031 (2021).
10. N. Zhang, S-SCAM, a rare copy number variation gene, induces schizophrenia-related endophenotypes in transgenic mouse model. *J Neurosci* **35**, 1892-1904 (2015).
11. H. Won *et al.*, Autistic-like social behaviour in Shank2-mutant mice improved by restoring NMDA receptor function. *Nature* **486**, 261-265 (2012).
12. X. Wang *et al.*, Synaptic dysfunction and abnormal behaviors in mice lacking major isoforms of Shank3. *Hum Mol Genet* **20**, 3093-3108 (2011).

13. H. Y. Koh, D. Kim, J. Lee, S. Lee, H. S. Shin, Deficits in social behavior and sensorimotor gating in mice lacking phospholipase C beta1. *Genes Brain Behav* **7**, 120-128 (2008).
14. Y. Yang, Ketamine blocks bursting in the lateral habenula to rapidly relieve depression. *Nature* **554**, 317-322 (2018).
15. D. H. Kim, B. R. Choi, W. K. Jeon, J. S. Han, Impairment of intradimensional shift in an attentional set-shifting task in rats with chronic bilateral common carotid artery occlusion. *Behav Brain Res* **296**, 169-176 (2016).
16. T. Kos, A. Nikiforuk, D. Rafa, P. Popik, The effects of NMDA receptor antagonists on attentional set-shifting task performance in mice. *Psychopharmacology (Berl)* **214**, 911-921 (2011).
17. K. Chisholm, A. Lin, A. Abu-Akel, S. J. Wood, The association between autism and schizophrenia spectrum disorders: A review of eight alternate models of co-occurrence. *Neurosci Biobehav Rev* **55**, 173-183 (2015).
18. I. Kushima *et al.*, Comparative Analyses of Copy-Number Variation in Autism Spectrum Disorder and Schizophrenia Reveal Etiological Overlap and Biological Insights. *Cell Rep* **24**, 2838-2856 (2018).
19. J. L. Doherty, M. J. Owen, Genomic insights into the overlap between psychiatric disorders: implications for research and clinical practice. *Genome Med* **6**, 29 (2014).
20. C. R. Marshall *et al.*, Contribution of copy number variants to schizophrenia from a genome-wide study of 41,321 subjects. *Nat Genet* **49**, 27-35 (2017).
21. A. Moreno-De-Luca *et al.*, Developmental brain dysfunction: revival and expansion of old concepts based on new genetic evidence. *Lancet Neurol* **12**, 406-414 (2013).
22. C. Y. RK *et al.*, Whole genome sequencing resource identifies 18 new candidate genes for autism spectrum disorder. *Nat Neurosci* **20**, 602-611 (2017).
23. L. Yu, Y. Wu, B. L. Wu, Genetic architecture, epigenetic influence and environment exposure in the pathogenesis of Autism. *Sci China Life Sci* **58**, 958-967 (2015).
24. C. S. Leblond *et al.*, Meta-analysis of SHANK Mutations in Autism Spectrum Disorders: a gradient of severity in cognitive impairments. *PLoS Genet* **10**, e1004580 (2014).
25. T. R. Mercer, M. E. Dinger, J. S. Mattick, Long non-coding RNAs: insights into functions. *Nat Rev Genet* **10**, 155-159 (2009).
26. P. Wu *et al.*, Roles of long noncoding RNAs in brain development, functional diversification and neurodegenerative diseases. *Brain Res Bull* **97**, 69-80 (2013).
27. B. R. Iyengar *et al.*, Non-coding RNA interact to regulate neuronal development and function. *Front Cell Neurosci* **8**, 47 (2014).

28. J. A. Briggs, E. J. Wolvetang, J. S. Mattick, J. L. Rinn, G. Barry, Mechanisms of Long Non-coding RNAs in Mammalian Nervous System Development, Plasticity, Disease, and Evolution. *Neuron* **88**, 861-877 (2015).
29. B. S. Clark, S. Blackshaw, Long non-coding RNA-dependent transcriptional regulation in neuronal development and disease. *Front Genet* **5**, 164 (2014).
30. Y. Wang *et al.*, Genome-wide differential expression of synaptic long noncoding RNAs in autism spectrum disorder. *Transl Psychiatry* **5**, e660 (2015).
31. M. N. Ziats, O. M. Rennert, Aberrant expression of long noncoding RNAs in autistic brain. *J Mol Neurosci* **49**, 589-593 (2013).
32. B. Wilkinson, D. B. Campbell, Contribution of long noncoding RNAs to autism spectrum disorder risk. *Int Rev Neurobiol* **113**, 35-59 (2013).
33. R. F. Wintle *et al.*, A genotype resource for postmortem brain samples from the Autism Tissue Program. *Autism Res* **4**, 89-97 (2011).
34. E. G. Puffenberger *et al.*, A homozygous missense mutation in HERC2 associated with global developmental delay and autism spectrum disorder. *Hum Mutat* **33**, 1639-1646 (2012).
35. G. V. Harlalka *et al.*, Mutation of HERC2 causes developmental delay with Angelman-like features. *J Med Genet* **50**, 65-73 (2013).
